# Explainable Stacked Ensemble Learning for Predicting Antibiotic Residues in the Danube River Within the Territory of the City of Novi Sad, Serbia

**DOI:** 10.3390/antibiotics15090920

**Published:** 2026-09-17

**Authors:** Dušan Kekić, Miloš Jovićević, Olja Šovljanski, Ana Tomić, Lato Pezo, Nemanja Mirković, Radmila Novaković, Ivan Vićić, Nikola Bajčetić, Ljiljana Tolić Stojadinović, Svetlana Grujić, Milica Mirković, Nedjeljko Karabasil, Nataša Opavski, Ina Gajić

**Affiliations:** 1 Institute of Microbiology and Immunology, Faculty of Medicine, University of Belgrade, 11000 Belgrade, Serbia; dusan.kekic@med.bg.ac.rs (D.K.); milos.jovicevic@med.bg.ac.rs (M.J.); natasa.vuckovic-opavski@med.bg.ac.rs (N.O.); ina.gajic@med.bg.ac.rs (I.G.); 2 Faculty of Technology Novi Sad, University of Novi Sad, 21000 Novi Sad, Serbia; oljasovljanski@uns.ac.rs; 3 Institute of General and Physical Chemistry, University of Belgrade, 11000 Belgrade, Serbia; latopezo@yahoo.co.uk; 4 Faculty of Agriculture, University of Belgrade, 11000 Belgrade, Serbia; nemanja.mirkovic@agrif.bg.ac.rs (N.M.); nikola.bajcetic@agrif.bg.ac.rs (N.B.); petrusicm@agrif.bg.ac.rs (M.M.); 5 Institute of Molecular Genetics and Genetic Engineering, University of Belgrade, 11000 Belgrade, Serbia; radmila.novakovic@imgge.bg.ac.rs; 6 Department of Food Hygiene and Technology, Faculty of Veterinary Medicine, University of Belgrade, 11000 Belgrade, Serbia; ivan.vicic@vet.bg.ac.rs (I.V.); nedja@vet.bg.ac.rs (N.K.); 7 Innovation Center of the Faculty of Technology and Metallurgy, University of Belgrade, 11000 Belgrade, Serbia; ljtolic@tmf.bg.ac.rs; 8 Faculty of Technology and Metallurgy, University of Belgrade, 11000 Belgrade, Serbia; svetlana.grujic@tmf.bg.ac.rs

**Keywords:** antibiotic contamination, surface waters, machine learning, stacked ensemble learning, random forest, explainable artificial intelligence, SHAP analysis, physicochemical parameters, microbial indicators, environmental monitoring

## Abstract

**Background/Objectives**: Antibiotic residues in aquatic environments reflect interacting physicochemical, climatic, and microbiological processes. This study characterized selected antibiotics in wastewater and surface water associated with the Danube River near Novi Sad, Serbia, and evaluated explainable stacked machine-learning models for concentration prediction. **Methods**: Thirty-six samples collected during summer and autumn 2024 were analyzed using SPE-HPLC-MS/MS. Artificial neural network, random forest, support vector machine, XGBoost, stacked linear, and stacked random forest (STACK-RF) models were developed using environmental/physicochemical variables or presumptive resistant bacterial taxa. Models were evaluated by fivefold cross-validation, complementary error metrics, Holm-adjusted Diebold–Mariano tests, XGBoost Gain, and SHAP analysis. **Results**: All target antibiotics were detected at least once. Azithromycin was most prevalent (75.0%), followed by sulfamethoxazole (58.3%), trimethoprim, and ciprofloxacin (52.8% each), while wastewater generally exhibited broader antibiotic profiles and higher concentrations than surface water. Standalone algorithms showed weak-to-moderate performance, whereas STACK-RF achieved the highest numerical accuracy for all environmental/physicochemical models (R^2^ = 0.745–0.913) and the available microbial-taxa models (R^2^ = 0.819–0.945), with consistently lower prediction errors. However, most pairwise differences were not significant after Holm correction. Influential environmental predictors were compound-specific and included COD, BOD_5_, pH, turbidity, electrical conductivity, water temperature, and relative humidity. Leading bacterial predictors included *Klebsiella pneumoniae*, *Escherichia coli*, *Citrobacter freundii*, and *Aeromonas veronii*. **Conclusions**: The results provide a proof-of-concept for machine-learning-assisted antibiotic prediction. Explainable STACK-RF modeling captured nonlinear, antibiotic-specific associations among residues, water-quality conditions, and microbial indicators. It may complement targeted chemical monitoring and support hypothesis generation, although larger, externally validated datasets are required before broader application.

## 1. Introduction

Antibiotics are indispensable for the prevention and treatment of bacterial infections in human and veterinary medicine; however, their extensive consumption has resulted in their continuous release into the environment. After administration, variable fractions of the active compounds are excreted unchanged or as biologically active metabolites and enter municipal wastewater systems. Additional inputs originate from hospitals, pharmaceutical manufacturing, livestock production, manure application, aquaculture, and the improper disposal of unused medicines. Because conventional wastewater treatment plants were not specifically designed to remove most pharmaceutical residues, antibiotics and their transformation products may pass into receiving rivers, lakes, sediments, and groundwater. A global survey covering 1052 sampling sites in 104 countries demonstrated that pharmaceutical contamination is geographically widespread and particularly pronounced in regions affected by inadequate wastewater infrastructure, intensive urbanization, and pharmaceutical or agricultural activities [1]. River-basin studies have likewise detected complex mixtures of fluoroquinolones, sulfonamides, tetracyclines, macrolides, β-lactams, and other antibiotic classes, often accompanied by clear spatial and seasonal variability [2,3]. Once introduced into aquatic ecosystems, the distribution of antibiotics is governed by interconnected physicochemical, hydrological, and biological processes. Compound-specific properties, including ionization state, hydrophobicity, aqueous solubility, sorption affinity, and susceptibility to hydrolysis or photodegradation, interact with environmental conditions such as pH, temperature, turbidity, electrical conductivity, organic matter content, water flow, and suspended-particle concentration. These interactions determine whether an antibiotic remains dissolved, adsorbs to sediment or particulate matter, undergoes transformation, or is transported downstream. Consequently, antibiotics belonging to different structural and therapeutic classes may exhibit markedly different environmental profiles even when released from the same source. For example, fluoroquinolones and tetracyclines generally display strong sorption to particles and sediment, whereas several sulfonamides and trimethoprim are comparatively mobile in the aqueous phase. Seasonal changes in river discharge, temperature, rainfall, wastewater dilution, and agricultural runoff further modify their concentrations and environmental persistence [1,2,3]. Antibiotic occurrence therefore reflects not only the magnitude of anthropogenic emissions, but also the local conditions controlling their transport and fate.

The environmental relevance of antibiotic residues extends beyond their direct ecotoxicological effects. Aquatic ecosystems contain diverse and highly interconnected microbial communities that may be exposed continuously to low or fluctuating antibiotic concentrations. Such exposure can alter the occurrence profiles of culture-based presumptive resistant bacterial taxa, metabolic activity, biodiversity, and ecological functions while also creating selective conditions that favor antibiotic-resistant bacteria and antibiotic resistance genes. Predicted no-effect concentrations for resistance selection have therefore been proposed as complementary benchmarks for environmental risk assessment [4]. Experimental evidence has shown that concentrations substantially below the minimum inhibitory concentrations used in clinical microbiology may enrich resistant populations or specific resistance determinants in complex aquatic biofilms [5]. Field investigations downstream of wastewater-treatment discharges have also revealed changes in antibiotic profiles, microbiome composition, resistomes, and mobile genetic elements, although the relationships among these components are frequently nonlinear and may be influenced by co-selective agents, nutrient enrichment, hydrological conditions, and the structure of the resident microbial community [6]. Large-scale microbiome and resistome analyses further indicate that wastewater effluents, landfill inputs, livestock-associated pollution, and densely populated areas can shape the environmental distribution of clinically relevant resistance determinants and their potential bacterial hosts [7]. These findings place antibiotic pollution within a One Health context in which chemical residues, environmental microorganisms, antimicrobial resistance, animal health, and human exposure are closely connected. The relationship between antibiotic concentrations and microbial indicators is nevertheless not straightforward. Antibiotics may modify microbial communities through selective pressure, but microbial populations can also influence antibiotic persistence through biodegradation, biotransformation, biosorption, and biofilm-mediated retention. Moreover, bacterial taxa detected in contaminated waters may reflect common pollution sources rather than a direct biological response to individual antibiotic compounds. Urban wastewater, hospital effluents, agricultural runoff, and animal waste can simultaneously introduce antibiotics, resistant bacteria, resistance genes, nutrients, organic matter, and fecal microorganisms. Accordingly, correlations between particular bacterial taxa and antibiotic concentrations may arise from shared sources, ecological interactions, differential tolerance, or environmental filtering. Studies of urban rivers have demonstrated that sewage inputs can generate distinct spatial and temporal patterns among resistance genes, while the strength of their relationships with physicochemical and microbiological variables differs among antibiotic classes [8].

Reliable monitoring of antibiotics in surface waters usually requires sensitive analytical techniques, particularly solid-phase extraction coupled with liquid chromatography-tandem mass spectrometry or high-resolution mass spectrometry. These methods remain essential for compound identification and quantitative confirmation, but extensive monitoring programs are constrained by analytical costs, specialized instrumentation, sample-preparation requirements, and the need to capture substantial spatial and temporal heterogeneity. Discrete sampling campaigns may therefore only provide limited snapshots of dynamic aquatic systems. Conventional statistical methods, including correlation analysis, multiple linear regression, and analysis of variance, are valuable for hypothesis testing and the identification of relatively simple relationships. However, their performance may be limited when predictor variables are numerous, collinear, interacting, non-normally distributed, or related to pollutant concentrations through nonlinear and threshold-dependent responses. These limitations are particularly relevant to antibiotic contamination, which is controlled by multiple simultaneous processes involving emission sources, water chemistry, climatic conditions, hydrology, particle transport, and microbial ecology.

Machine learning provides a complementary data-driven approach for extracting predictive information from such multidimensional environmental datasets. Algorithms including artificial neural networks (ANN), random forests (RF), support vector machines (SVM), and Extreme Gradient Boosting (XGBoost) can accommodate nonlinear relationships and higher-order interactions without requiring the response to follow a predetermined analytical function. Their applications in aquatic research include the prediction of water-quality indices, nutrient concentrations, turbidity, algal abundance, pollutant distributions, microbial activity, and ecological status. Nevertheless, the use of machine learning does not eliminate the need for methodological rigor. Inadequate sample size, data leakage, unrepresentative train–test partitioning, excessive predictor dimensionality, insufficient hyperparameter optimization, and inappropriate performance metrics can produce overly optimistic results and weak external generalizability [9]. Cross-validation, transparent preprocessing, comparison of structurally different algorithms, and evaluation using multiple complementary error measures are therefore essential, particularly when environmental datasets contain relatively few samples.

Direct applications of machine learning to antibiotic concentration prediction remain comparatively uncommon. A recent study demonstrated that ciprofloxacin concentrations could be estimated from conventional water-quality indicators, including chemical oxygen demand, ammonium nitrogen, dissolved oxygen, water temperature, total nitrogen, and pH, using optimized machine-learning algorithms [10]. This result supports the premise that routinely measured environmental variables can contain indirect information on antibiotic inputs, dilution, transport, and persistence. However, a single algorithm may capture only part of the underlying data structure. ANN models are capable of representing complex functional relationships but can be sensitive to architecture, initialization, and sample size. SVM models can perform effectively in high-dimensional spaces but depend strongly on kernel and parameter selection. RF models are comparatively robust to nonlinearities and interactions but may smooth extreme responses, whereas XGBoost can provide high predictive accuracy but is susceptible to overfitting when model complexity is not appropriately controlled. Comparative evaluation is therefore preferable to selecting an algorithm a priori. Ensemble learning can further improve prediction by combining complementary information from multiple base learners. In stacking, predictions generated by individual models are supplied to a higher-level meta-learner, which determines how the component predictions should be combined [11,12]. This structure may reduce model-specific bias and variance when the base algorithms make partly independent errors. Stacking approaches have produced encouraging results in surface-water modeling, including the prediction of individual water-quality parameters and composite water-quality indices [13,14]. However, high numerical accuracy alone is insufficient for environmental interpretation. Complex ensemble and boosting models are frequently treated as “black boxes”, making it difficult to determine whether their predictions are based on scientifically plausible relationships or dataset-specific artifacts. Furthermore, improvements in descriptive metrics should not automatically be interpreted as statistically confirmed model superiority, particularly when sample sizes are limited or multiple algorithms are compared. Explainable artificial intelligence offers a means of addressing this limitation. SHapley Additive exPlanations (SHAP) assign each predictor a contribution to an individual model output and allow these local contributions to be aggregated into global importance patterns [15]. In contrast to rankings based solely on split frequency or impurity reduction, SHAP can provide information on both the magnitude and direction of predictor effects. Explainable machine-learning frameworks have been successfully applied to identify the environmental controls of spatial water-quality heterogeneity, algal dynamics, river pollution, and microbial activity [11,12]. Park et al. [12], for example, demonstrated that ensemble predictions of water quality could be interpreted by combining SHAP values with conventional feature-importance and multicollinearity analyses. More recently, stacked regression and SHAP-based frameworks have been used to identify dissolved oxygen, biochemical oxygen demand, electrical conductivity, and pH as influential determinants of water-quality-index predictions [14]. These applications illustrate how explainability can transform machine learning from a purely predictive tool into a hypothesis-generating approach capable of identifying environmental variables that warrant further mechanistic investigation. Nevertheless, SHAP values describe the contribution of variables to a fitted model and should not, in isolation, be interpreted as evidence of causality [9,15].

Despite these advances, important knowledge gaps remain. Most machine-learning studies in aquatic systems have focused on general water-quality indicators, nutrients, algal blooms, or individual contaminants. Studies directly predicting multiple antibiotics are scarce, and available investigations commonly consider only the physicochemical predictors or a single antibiotic. Conversely, microbial modeling studies have demonstrated that environmental and water-quality variables can predict microbial activity, abundance, and diversity [13,16], but the reciprocal use of bacterial profiles as predictors of antibiotic concentrations has received much less attention. Moreover, few studies have systematically compared individual algorithms with stacked meta-learning approaches while also applying explainability methods to distinguish compound-specific predictor patterns. Evaluating physicochemical and microbiological information in parallel may clarify whether antibiotic concentrations are associated primarily with immediate hydrochemical conditions, broader pollution-source signatures, microbial-community structure, or combinations of these factors [17].

Accordingly, the present study aimed to develop and compare ANN, RF, SVM, XGBoost, a stacked linear model (STACK-LM), and a stacked random forest model (STACK-RF) for predicting the concentrations of selected environmentally relevant antibiotics (trimethoprim, ciprofloxacin, amoxicillin, ceftriaxone, sulfamethoxazole, azithromycin, and doxycycline) in surface water and wastewater samples. Two predictor domains were evaluated: environmental and physicochemical variables, and the occurrence profiles of selected bacterial species. Predictive performance was examined using fivefold cross-validation and multiple complementary statistical criteria, while pairwise differences in forecast accuracy were further assessed to avoid relying exclusively on descriptive performance rankings. Feature-importance measures and SHAP analysis were used to identify the variables contributing most strongly to the prediction of each antibiotic concentration. By combining comparative model evaluation, ensemble learning, and explainable artificial intelligence, this study provides a proof-of-concept framework for examining nonlinear associations among antibiotic occurrence and local environmental conditions.

A limitation of the present study is the absence of an independent external validation dataset. Although fivefold cross-validation provides an efficient approach for estimating predictive performance when the available sample size is limited, it evaluates model behavior primarily within the environmental, spatial, and temporal domain represented by the observed samples. Consequently, the present results do not establish transferability to unobserved hydrological conditions, future monitoring periods, or geographically distinct river systems. External validation using independently collected spatial and temporal datasets is therefore required before the developed models can be considered suitable for routine monitoring applications. Future studies should include year-round, multi-year sampling at multiple locations, and where possible, validation across geographically distinct aquatic systems to assess model transportability and robustness under broader environmental conditions.

## 2. Results

### 2.1. Occurrence of Antibiotics in Wastewater and Surface-Water Samples

**Figure 1:**
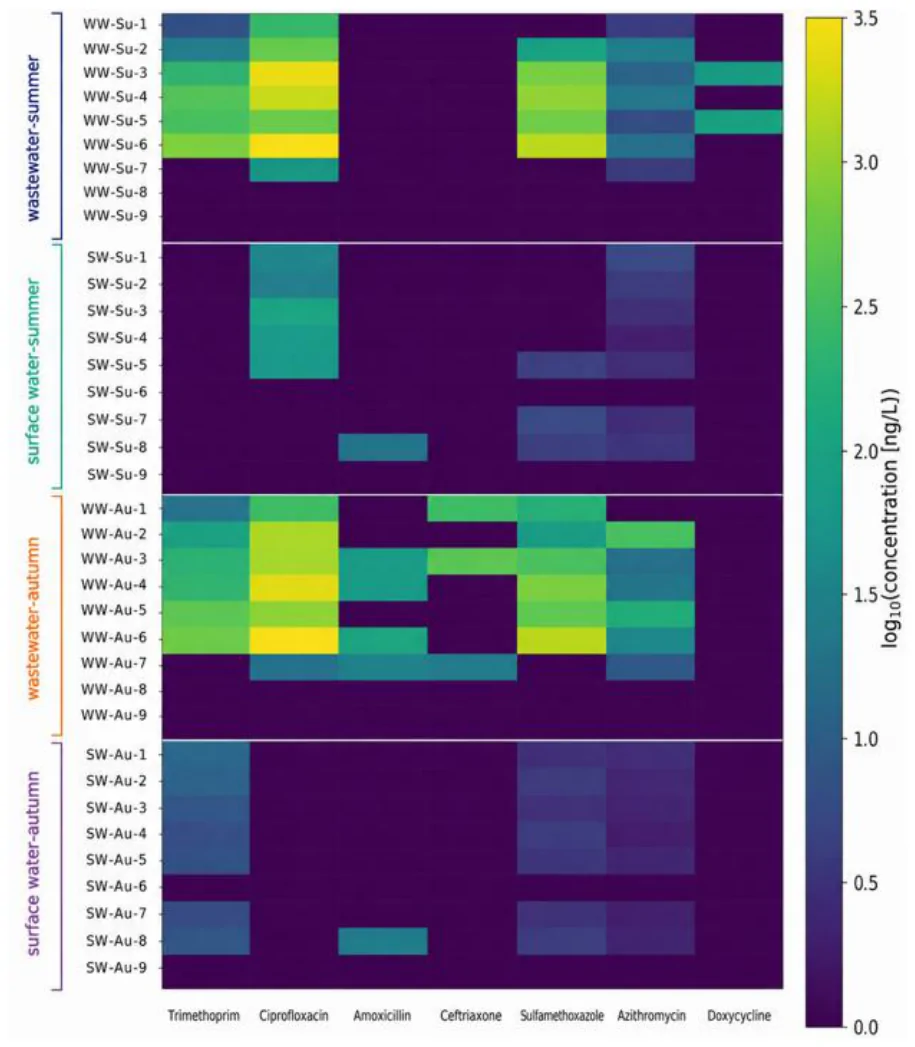
Heatmap of antibiotic concentration profiles in 36 samples. Values were log10-transformed after adding 1 for visualization. WW, wastewater; SW, surface water; Su, summer; Au, autumn. Sequential numbers indicate the row order in the supplied dataset.

All target antibiotics were detected at least once among the 36 analyzed samples, although their occurrence differed markedly by water matrix and season (Table 1 and Table S1, Figure 1). Azithromycin was the most frequently detected compound (27/36 samples, 75.0%), followed by sulfamethoxazole (21/36, 58.3%), trimethoprim and ciprofloxacin (both 19/36, 52.8%), amoxicillin (6/36, 16.7%), ceftriaxone (3/36, 8.3%), and doxycycline (2/36, 5.6%). For the descriptive occurrence analysis, zero entries in the supplied analytical dataset were treated as non-detections.

Wastewater samples showed both broader antibiotic profiles and substantially higher reported concentrations than surface water. Ciprofloxacin, azithromycin, trimethoprim, and sulfamethoxazole were detected in 77.8%, 72.2%, 66.7%, and 61.1% of wastewater samples, respectively. The highest reported concentrations were recorded in wastewater for ciprofloxacin (autumn), sulfamethoxazole (summer), trimethoprim (summer), ceftriaxone (autumn), azithromycin (autumn), doxycycline (summer), and amoxicillin (autumn). In contrast, ceftriaxone and doxycycline were not detected in any surface-water sample. Seasonal patterns in surface water were compound-specific. Trimethoprim was absent in summer but detected in seven out of nine autumn samples, whereas ciprofloxacin was detected in five out of nine summer samples and was absent in autumn. Sulfamethoxazole detection increased from three out of nine summer samples to seven out of nine autumn samples, while azithromycin was detected in seven out of nine samples in both seasons. In wastewater, amoxicillin and ceftriaxone were detected only in autumn, whereas doxycycline was detected only in summer.

### 2.2. Predictive Performance Using Environmental and Physicochemical Variables

**Figure 2:**
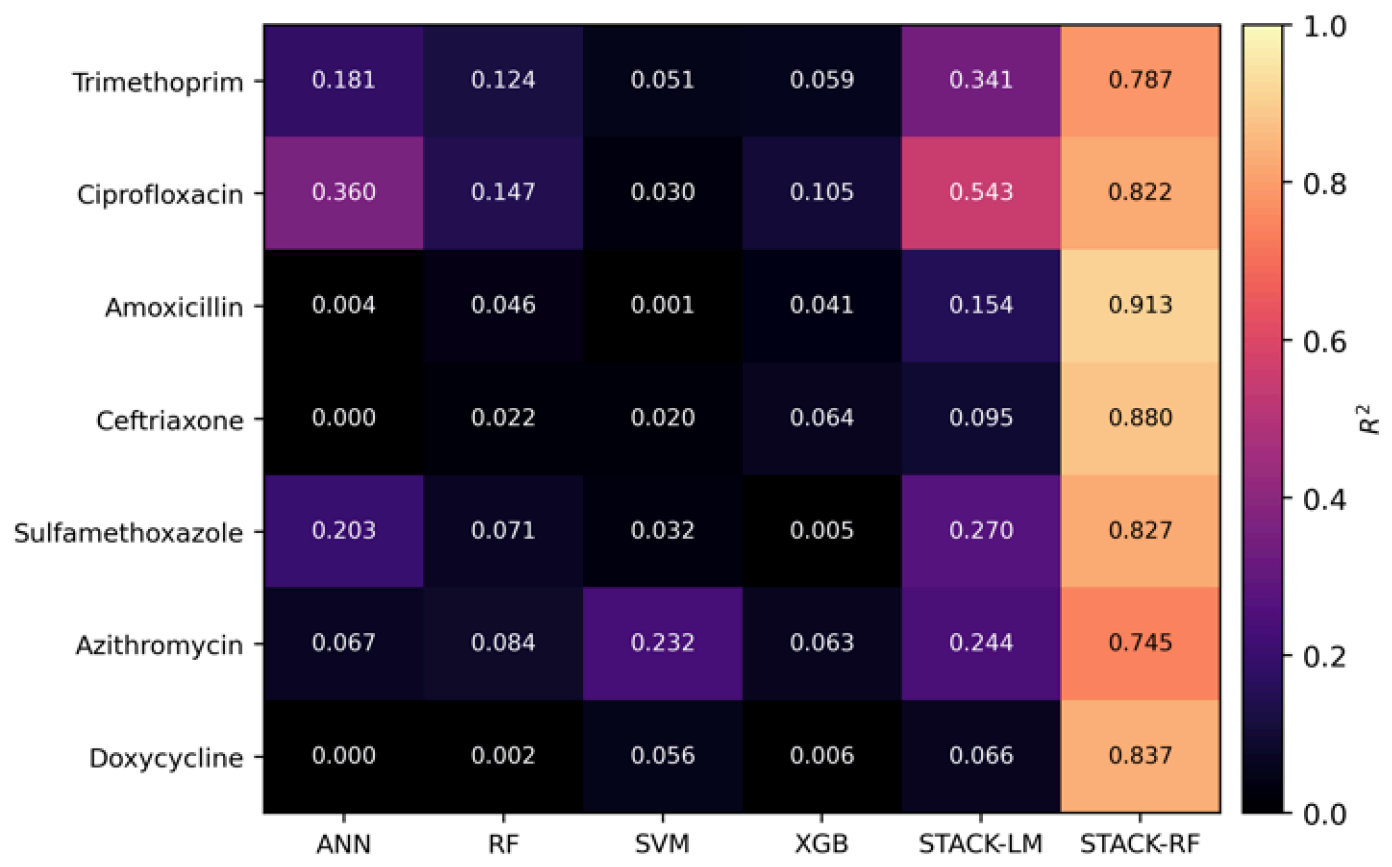
Heatmap of fivefold cross-validated R^2^ values for the six machine-learning approaches across the seven antibiotics for environmental and physicochemical predictor models (STACK-LM, stacked linear meta-model; STACK-RF, stacked random forest meta-model).

The predictive performance of the six evaluated modeling approaches differed substantially across algorithms and antibiotics (Table 2, Figure 2). The four standalone models generally showed weak to moderate performance, with R^2^ values ranging from 0.000 to 0.360. Among the individual algorithms, ANN produced the highest R^2^ for trimethoprim (0.181), ciprofloxacin (0.360), and sulfamethoxazole (0.203); RF was numerically best for amoxicillin (0.046); XGBoost was best for ceftriaxone (0.064); and SVM performed best for azithromycin (0.232) and doxycycline (0.056). The linear stacking model improved R^2^ relative to the best standalone model for all seven antibiotics, but its performance remained limited for amoxicillin, ceftriaxone, and doxycycline (R^2^ = 0.154, 0.095, and 0.066, respectively). The STACK-RF model achieved the highest numerical accuracy for every antibiotic, with R^2^ values of 0.787 for trimethoprim, 0.822 for ciprofloxacin, 0.913 for amoxicillin, 0.880 for ceftriaxone, 0.827 for sulfamethoxazole, 0.745 for azithromycin, and 0.837 for doxycycline. These improvements were accompanied by the lowest RMSE, MAE, and SSE values within each antibiotic-specific comparison.

The ensemble stacking approach based on random forest (STACK-RF) consistently provided the most favorable numerical predictive performance out of all individual models and the linear stacking approach, achieving the highest coefficient of determination (R^2^) and the lowest prediction errors for all antibiotic concentrations investigated. The prediction of trimethoprim concentration showed moderate performance for individual models (R^2^ = 0.051–0.181), whereas STACK-RF markedly improved predictive accuracy (R^2^ = 0.787, RMSE = 100.64, MAE = 64.26, SSE = 3.6 × 10^5^), indicating that approximately 78.7% of the variability in trimethoprim concentration could be explained by the combined ensemble model. A similar trend was observed for ciprofloxacin, where individual models explained only 3.0–36.0% of the variance, while STACK-RF achieved R^2^ = 0.822 with substantially reduced RMSE (313.44) and MAE (186.30). The greatest improvement was obtained for amoxicillin, for which individual models exhibited almost negligible predictive power (R^2^ ≤ 0.046), whereas STACK-RF reached R^2^ = 0.913 and reduced SSE to 5.5 × 10^3^, demonstrating high numerical cross-validated performance, which should be interpreted cautiously because of the sparse positive detections. Likewise, ceftriaxone concentration prediction improved from very weak individual-model performance (R^2^ ≤ 0.064) to R^2^ = 0.880 with STACK-RF, accompanied by the lowest RMSE (35.57) and MAE (14.05). For sulfamethoxazole, individual algorithms explained only 0.5–20.3% of the variance, whereas STACK-RF achieved R^2^ = 0.827, reducing prediction errors by approximately three- to fivefold relative to the weakest models. Azithromycin exhibited moderate predictability, with the SVM model performing best among individual algorithms (R^2^ = 0.232), but STACK-RF substantially increased the predictive performance to R^2^ = 0.745 while reducing RMSE to 29.40 and MAE to 12.80. Finally, doxycycline proved difficult to predict using individual models (R^2^ ≤ 0.066), yet STACK RF achieved R^2^ = 0.837 and reduced SSE to 8.7 × 10^3^. In contrast, the individual ANN, RF, SVM, and XGB models generally demonstrated low to moderate predictive performance, suggesting that the relationships between environmental variables and antibiotic concentrations are highly nonlinear and complex.

The favorable performance of the STACK RF ensemble indicates that integrating concentration prediction from multiple machine-learning algorithms effectively captures complementary patterns within the ecological and physicochemical dataset, leading to substantial improvements in predictive accuracy and robustness across all antibiotic classes examined.

Before correction for multiple comparisons, several Diebold–Mariano contrasts yielded nominal *p*-values below 0.05, particularly in comparisons involving STACK-RF for trimethoprim, ciprofloxacin, amoxicillin, and sulfamethoxazole. After Holm adjustment, however, most pairwise differences were no longer statistically significant (Table 3). Only two amoxicillin-related contrasts remained significant after correction: SVM versus XGBoost (adjusted *p* = 0.049) and XGBoost versus STACK-RF (adjusted *p* = 0.039). No adjusted pairwise comparison was significant for trimethoprim, ciprofloxacin, ceftriaxone, sulfamethoxazole, azithromycin, or doxycycline. Thus, the descriptive performance metrics consistently favored STACK-RF, but statistically strong evidence of unequal predictive accuracy was restricted to the two amoxicillin comparisons.

Prior to correction for multiple comparisons, several pairwise contrasts suggested favorable performance of the STACK-RF model relative to individual models, particularly for trimethoprim, ciprofloxacin, amoxicillin, and sulfamethoxazole, where positive DM statistics (DM = 2.06–3.25) and nominal *p*-values below 0.05 indicated lower forecast errors for the ensemble approach. However, after applying the Holm correction to control the family-wise error rate, most pairwise differences were no longer statistically significant (adjusted *p* > 0.05), indicating that the apparent superiority observed in unadjusted comparisons should be interpreted cautiously.

The strongest evidence of differential predictive performance was observed for amoxicillin, where comparisons involving XGB versus SVM (Holm-adjusted *p* = 0.049) and XGB versus STACK-RF (Holm-adjusted *p* = 0.039) remained statistically significant, demonstrating meaningful differences in predictive accuracy among these models. For trimethoprim and ciprofloxacin, although STACK-RF consistently exhibited the highest predictive performance according to the R^2^, RMSE, MAE, and SSE metrics, none of the pairwise DM comparisons remained significant after Holm adjustment (adjusted *p* ≥ 0.112). Similarly, for sulfamethoxazole, several nominally significant comparisons involving STACK-RF and XGB were observed (unadjusted *p* = 0.004–0.029), but significance was lost after correction (Holm-adjusted *p* ≥ 0.060). For ceftriaxone, azithromycin, and doxycycline, all pairwise comparisons were non-significant following adjustment (Holm-adjusted *p* = 0.579–1.000), indicating that the predictive performances of the competing algorithms could not be statistically distinguished despite differences in descriptive performance metrics. The DM analysis suggests that although ensemble-based approaches, particularly STACK-RF, generally achieved favorable predictive accuracy according to conventional performance indicators, the majority of observed differences among models were not statistically significant after controlling for multiple testing. This finding indicates that the predictive capabilities of the evaluated machine-learning methods were broadly comparable for most antibiotics, with a statistically favorable model detected only for a limited number of amoxicillin-related comparisons.

The supplied XGBoost feature-importance output showed distinct, antibiotic-specific predictor patterns (Table 4). Trimethoprim was primarily associated with chemical oxygen demand (COD; Gain = 0.484) and pH (0.287), whereas ciprofloxacin was dominated by turbidity (0.673) and electrical conductivity (0.195). Amoxicillin displayed a more balanced importance profile involving pH (0.255), water temperature (0.251), and relative air humidity (0.248). Relative air humidity was the dominant reported predictor for ceftriaxone (0.612). Sulfamethoxazole was most strongly associated with turbidity (0.576), followed by pH (0.199) and COD (0.109). Azithromycin was linked primarily to biochemical oxygen demand (BOD_5_; 0.311), COD (0.273), and relative air humidity (0.185), while doxycycline was dominated by electrical conductivity (0.681) and water temperature (0.267). Chemical and biological oxygen demand, pH, turbidity, electrical conductivity, water temperature, and relative air humidity were among the most influential predictors, with compound-specific effects. Site and sampling time generally exhibited lower predictive contributions within the temporal and spatial range represented by the available dataset.

The SHAP analysis of the XGBoost models revealed that the relative importance of ecological and physicochemical variables differed substantially among antibiotics, indicating compound-specific environmental controls on antibiotic occurrence. For trimethoprim, COD was the dominant predictor (Gain = 0.484), followed by pH (Gain = 0.287), whereas pressure, turbidity, and air temperature contributed considerably less, suggesting that organic pollution and acid–base conditions were the primary determinants of trimethoprim variability. Ciprofloxacin concentrations were predominantly influenced by turbidity (Gain = 0.673), with electrical conductivity (Gain = 0.195) representing the second most important predictor, indicating a strong association with suspended particles and dissolved ionic constituents. For amoxicillin, pH (Gain = 0.255), water temperature (Gain = 0.251), and relative air humidity (Gain = 0.248) exhibited comparable contributions, suggesting that both hydrochemical and climatic conditions influenced its distribution. For ceftriaxone, relative air humidity had the highest XGBoost Gain value (0.612), indicating its relative contribution to predictions within this fitted model. This finding does not establish a direct effect of atmospheric humidity on ceftriaxone concentrations. Ceftriaxone was detected in only three samples, all collected from wastewater during the autumn campaign; therefore, the importance assigned to humidity may reflect differences between sampling campaigns or characteristics of these few positive observations. This predictor ranking should consequently be regarded as exploratory. Sulfamethoxazole was primarily controlled by turbidity (Gain = 0.576), followed by pH (Gain = 0.199) and COD (Gain = 0.109), highlighting the importance of particulate matter and organic load. For azithromycin, BOD_5_ represented the most influential predictor (Gain = 0.311), followed by COD (Gain = 0.273) and relative air humidity (Gain = 0.185), suggesting a close relationship between azithromycin occurrence and organic pollution indicators. In contrast, doxycycline concentrations were dominated by electrical conductivity (Gain = 0.681), with water temperature representing the second most influential factor (Gain = 0.267), whereas all other predictors exhibited relatively minor effects. Overall, the SHAP results demonstrated that water quality parameters, particularly COD, BOD_5_, pH, turbidity, electrical conductivity, and water temperature, were consistently among the most influential predictors across antibiotics, whereas spatial (Site) and temporal (Time) variables generally exhibited low importance, indicating that local physicochemical conditions exerted a substantially stronger influence on antibiotic concentrations than sampling location or season.

The variable importance analysis of the STACK-RF ensemble models further confirmed the favorable predictive contribution of the integrated machine-learning framework. The highest IncNodePurity values were observed for ciprofloxacin (2.75–3.60 × 10^6^), followed by sulfamethoxazole (6.64–7.63 × 10^5^), trimethoprim (2.14–2.84 × 10^5^), ceftriaxone (1.69–5.21 × 10^4^), azithromycin (1.36–1.68 × 10^4^), doxycycline (2.49–9.86 × 10^3^), and amoxicillin (2.98–7.57 × 10^3^). These findings are consistent with the predictive performance metrics, where STACK-RF achieved the highest coefficients of determination (R^2^ = 0.745–0.913) and the lowest prediction errors across all antibiotics. Collectively, the SHAP and ensemble importance analyses indicate that antibiotic occurrence in aquatic environments is governed by complex and antibiotic-specific interactions among hydrochemical conditions, organic pollution indicators, and climatic variables, while ensemble learning approaches are capable of effectively capturing these nonlinear relationships.

### 2.3. Predictive Performance Using Presumptive Resistant Bacterial Taxa Detected in Wastewater and Surface Water Samples

**Figure 3:**
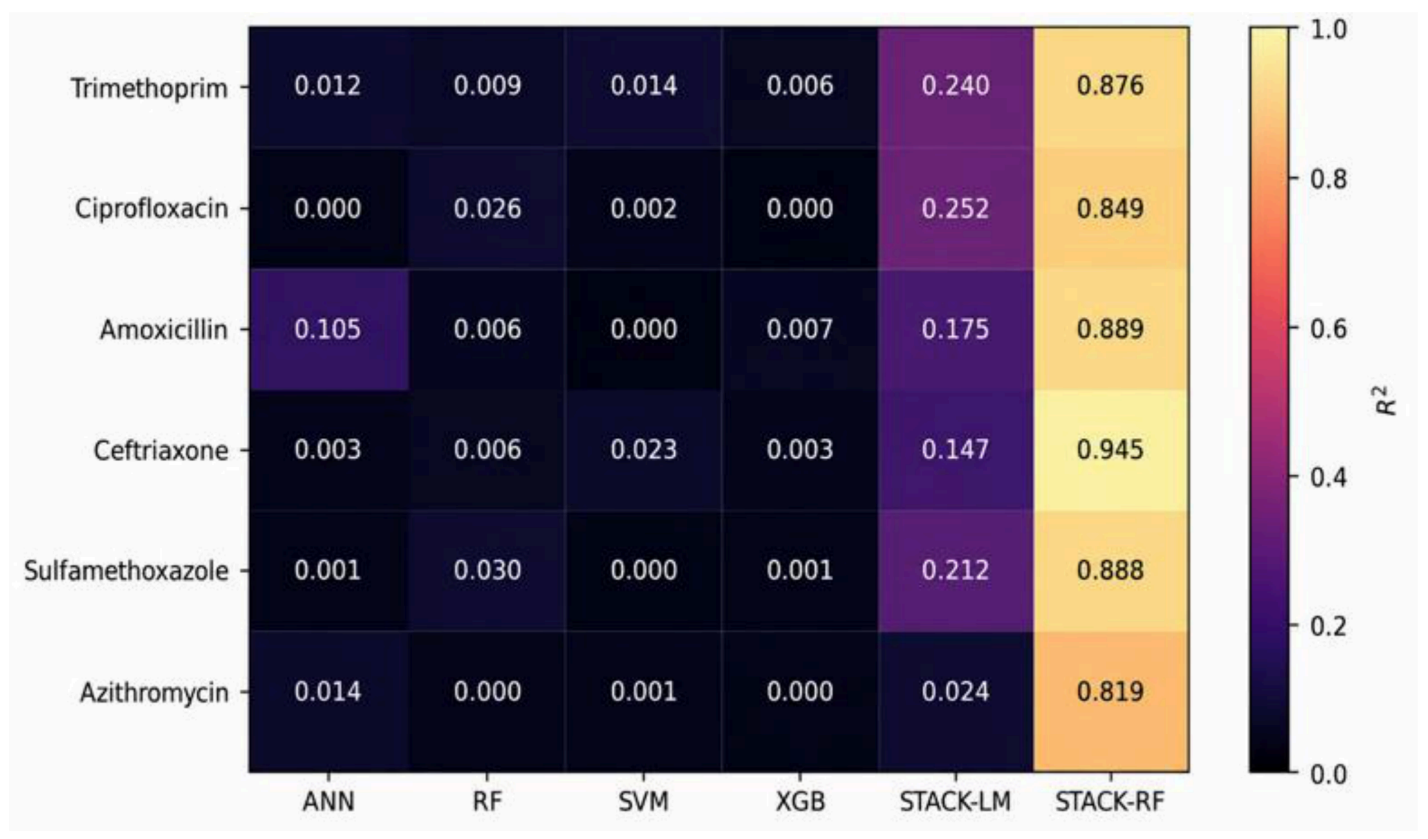
Heatmap of fivefold cross-validated R^2^ values for the six machine-learning approaches across the seven antibiotics for environmental and physicochemical predictor models (STACK-LM, stacked linear meta-model; STACK-RF, stacked random forest meta-model).

For the prediction of antibiotic concentrations based on the occurrence profiles of culture-based presumptive resistant bacterial taxa reported in the work of Jovićević et al. [18], the machine-learning models exhibited markedly different predictive capabilities, indicating varying degrees of association between microbial abundance patterns and antibiotic occurrence in the aquatic environment. The results are presented in Table 5 and Figure 3. The presented results demonstrate substantial differences in predictive performance among the evaluated machine-learning models. The individual models (ANN, RF, SVM, and XGB) exhibited limited predictive capability for antibiotic concentrations, as indicated by consistently low coefficients of determination (R^2^ ≤ 0.105), suggesting that these algorithms explained only a small fraction of the variability in the experimental data. In contrast, the stacking approaches, particularly the STACK-RF ensemble, consistently provided the most favorable numerical predictive performance all individual models across all antibiotics, achieving the highest R^2^ values together with the lowest prediction errors (RMSE, MAE, AARD, and SSE). For trimethoprim concentration prediction, the STACK-RF model achieved the highest predictive accuracy (R^2^ = 0.876), substantially improving upon the best individual model (SVM, R^2^ = 0.014).

This improvement was accompanied by the lowest RMSE (91.650), MAE (59.635), AARD (2.0 × 10^12^), and SSE (3.0 × 10^5^), indicating a marked reduction in prediction error relative to the remaining models. Similarly, for ciprofloxacin, STACK-RF explained 84.9% of the variance (R^2^ = 0.849), whereas the individual algorithms showed virtually no predictive capability (R^2^ ≤ 0.026). The ensemble also reduced the RMSE from approximately 683–870 to 369.743 and decreased SSE to 4.9 × 10^6^, representing the best overall performance. A comparable trend was observed for amoxicillin concentration prediction, where STACK-RF yielded the highest R^2^ (0.889) and the lowest RMSE (14.447), MAE (10.002), AARD (5.3 × 10^11^), and SSE (7.5 × 10^3^). The individual models explained less than 11% of the variance, with ANN providing the highest R^2^ (0.105) among them. For ceftriaxone, STACK-RF demonstrated excellent predictive capability (R^2^ = 0.945), considerably exceeding the performance of all other models (R^2^ ≤ 0.147) while simultaneously producing the smallest prediction errors (RMSE = 31.913, MAE = 13.564, AARD = 6.7 × 10^11^, and SSE = 3.7 × 10^4^).

In the present dataset, non-detections were represented as zero values; however, such observations should be interpreted as concentrations below the analytical detection capability. This treatment is particularly relevant for relative-error measures because AARD involves division by the observed concentration and can therefore become undefined or disproportionately large for zero or near-zero observations. Accordingly, AARD was interpreted only as a supplementary metric, whereas R^2^, RMSE, MAE, and SSE were used as the principal indicators of predictive performance. Future studies with larger datasets should incorporate detection limits explicitly and consider two-part or hurdle-type modeling, in which detection probability and concentration conditional on detection are modeled separately, or appropriately defined log-scale error measures.

For sulfamethoxazole concentration prediction, the favorability of the stacking ensemble remained evident. STACK-RF achieved an R^2^ of 0.888 compared with a maximum of only 0.212 for STACK-LM and 0.030 for the individual models. Correspondingly, RMSE, MAE, and SSE were reduced to 170.358, 118.525, and 1.0 × 10^6^, respectively, representing the most accurate predictions among the evaluated approaches. Likewise, for azithromycin concentration prediction, STACK-RF produced the highest coefficient of determination (R^2^ = 0.819) while reducing RMSE to 30.551, MAE to 15.797, AARD to 2.3 × 10^11^, and SSE to 3.4 × 10^4^. The remaining models showed negligible predictive ability (R^2^ ≤ 0.024) despite relatively moderate absolute errors for some algorithms.

The Diebold–Mariano (DM) test was used to compare the predictive accuracy of the six machine-learning models for each antibiotic (Table 6). The DM test with Holm-adjusted *p*-values was used to determine whether differences in predictive accuracy between pairs of machine-learning models were statistically significant after correcting for multiple comparisons. Overall, although the performance metrics demonstrated the clear favorability of the STACK-RF model, the pairwise statistical comparisons revealed that these improvements were generally not statistically significant after Holm correction, indicating that the observed gains should be interpreted with caution given the variability of the prediction errors.

For trimethoprim concentration prediction, none of the pairwise comparisons remained statistically significant after Holm adjustment (Holm-adjusted *p* > 0.05). Nevertheless, the largest positive DM statistics were consistently obtained for comparisons involving STACK-RF, particularly against XGB (DM = 3.091, adjusted *p* = 0.058), suggesting a strong trend toward favorable predictive accuracy that narrowly missed statistical significance. Similarly, comparisons between STACK-LM and XGB (DM = 2.388, adjusted *p* = 0.270) indicated improved performance of the stacking approach but without sufficient statistical evidence after correction. For ciprofloxacin concentration prediction, the only statistically significant difference after Holm adjustment was observed between STACK-RF and ANN (DM = 3.261, Holm-adjusted *p* = 0.037), confirming that the STACK-RF ensemble provided significantly more accurate predictions than the ANN model. All remaining comparisons yielded adjusted *p*-values exceeding 0.05, although positive DM statistics for STACK-RF relative to RF, SVM, XGB, and STACK-LM indicated a consistent tendency toward improved forecasting performance. For amoxicillin concentration prediction, no statistically significant pairwise differences were detected after Holm correction despite relatively large positive DM statistics for STACK-RF compared with XGB (DM = 3.101, adjusted *p* = 0.057), suggesting a borderline improvement in predictive accuracy. Likewise, comparisons involving STACK-LM and XGB (DM = 2.133) were not statistically significant following adjustment. For ceftriaxone concentration prediction, none of the pairwise comparisons reached statistical significance, with all Holm-adjusted *p*-values equal to 1.000. Although STACK-RF achieved the highest predictive performance according to the regression metrics, the DM analysis indicates that its prediction errors were not statistically distinguishable from those of the remaining models, likely reflecting the limited sample size and variability in forecast errors.

For sulfamethoxazole concentration prediction, the strongest statistical evidence favoring the ensemble approach was observed. The comparison between STACK-RF and XGB remained statistically significant after Holm adjustment (DM = 3.458, Holm-adjusted *p* = 0.022), demonstrating significantly greater predictive accuracy of the STACK-RF model. Other comparisons involving STACK-RF produced positive DM statistics (DM = 2.256–2.973), but the corresponding adjusted *p*-values exceeded the 0.05 significance threshold, indicating only trends toward improved performance. For azithromycin concentration prediction, no statistically significant differences were identified after multiple-testing correction. Nevertheless, positive DM statistics for STACK-RF relative to ANN (DM = 1.962), RF (DM = 1.460), and XGB (DM = 2.308) were consistent with the favorable regression performance reported in Table 6, although the observed improvements did not reach statistical significance. Finally, for doxycycline concentration prediction, all pairwise comparisons were non-significant after Holm adjustment (all adjusted *p* = 1.000). The relatively small DM statistics (|DM| ≤ 1.758) indicate that the prediction errors of the evaluated models were statistically comparable for this antibiotic.

The Diebold–Mariano analysis provided more limited statistical evidence for differences between models. Following Holm correction for multiple comparisons, most pairwise differences in predictive accuracy were not statistically significant. Thus, although STACK-RF was identified as the best-performing model according to the descriptive performance metrics, its numerical advantage should not be interpreted as consistent statistical superiority over the competing approaches.

XGBoost variable-importance analysis based on Gain revealed marked antibiotic-specific differences in the contribution of presumptive resistant bacterial taxa (Table 7). For trimethoprim, the leading microbial predictors were *Aeromonas veronii* (Gain = 0.128) and *Escherichia coli* (0.125), followed by *Citrobacter freundii* (0.081) and *Klebsiella pneumoniae* (0.058). Ciprofloxacin concentrations were most strongly associated with *C. freundii* (0.136), while *E. coli* (0.080), *Stenotrophomonas maltophilia* (0.078), and *A. veronii* (0.076) also contributed substantially to the model. For amoxicillin, *K. pneumoniae* was the dominant bacterial predictor (0.253), followed by *Citrobacter braakii* (0.107) and *S. maltophilia* (0.093). The ceftriaxone model showed the most strongly concentrated importance profile, with *K. pneumoniae* accounting for the highest Gain value (0.519), followed by *Acinetobacter baumannii* (0.321) and *Serratia marcescens* (0.150). For sulfamethoxazole, the principal bacterial predictors were *K. pneumoniae* (0.149), *A. veronii* (0.105), and *C. freundii* (0.101). In the azithromycin model, *E. coli* exhibited the highest bacterial-taxon importance (0.277).

Across the evaluated antibiotics, *K. pneumoniae*, *E. coli*, *C. freundii*, and *A. veronii* repeatedly appeared among the leading predictors, indicating that several wastewater-associated and clinically relevant bacterial taxa contributed to the observed antibiotic-specific prediction patterns. Nevertheless, these Gain values represent relative importance within the corresponding XGBoost models and should not be interpreted as evidence of causality. Moreover, given the generally low predictive performance of the standalone XGBoost models, the identified rankings should primarily be regarded as exploratory indicators of potential associations between bacterial community composition and antibiotic occurrence.

The SHAP-based variable importance analysis of the XGBoost models revealed that the relative contribution of microbial taxa and environmental variables differed considerably among the six antibiotics (Table 7). Feature importance was evaluated using Gain, which quantifies the improvement in model performance attributable to a variable, together with Cover, reflecting the proportion of observations influenced by that variable, and Frequency, indicating how often a variable was selected during tree construction. Overall, sampling site (Site R), sampling period (Time October), and several clinically relevant bacterial taxa, particularly *K. pneumoniae*, *E. coli*, *C. freundii*, *A. veronii*, and *S. marcescens*, consistently emerged as the most influential predictors, whereas numerous *Pseudomonas* species and less prevalent taxa exhibited negligible contributions. For trimethoprim concentration prediction, Site R was the dominant predictor (Gain = 0.326), indicating that spatial variation represented the strongest determinant of model predictions. The remaining influential variables included *A. veronii* (Gain = 0.128), *E. coli* (0.125), *C. freundii* (0.081), and Time October (0.066), suggesting that both microbial composition and seasonal variability contributed to trimethoprim occurrence. Although *K. pneumoniae* exhibited moderate Gain (0.058), it demonstrated relatively high Cover (0.126), indicating a broader influence across observations. For ciprofloxacin, Site R again represented the most influential variable (Gain = 0.370), exceeding all microbial predictors. Among the bacterial taxa, *C. freundii* (0.136), *E. coli* (0.080), *Stenotrophomonas maltophilia* (0.078), and *A. veronii* (0.076) contributed substantially to the model, whereas Time October exhibited only moderate importance (Gain = 0.030). These findings indicate that both spatial heterogeneity and the abundance of common wastewater-associated microorganisms were major determinants of ciprofloxacin concentrations. In contrast, amoxicillin concentration predictions were primarily driven by microbial variables rather than environmental descriptors. The highest importance was assigned to *K. pneumoniae* (Gain = 0.253), followed by Time October (0.183), *Citrobacter braakii* (0.107), and *S. maltophilia* (0.093). Notably, Site R ranked considerably lower (Gain = 0.041), suggesting that temporal variation and specific bacterial taxa explained a larger proportion of variability than spatial location. A markedly different pattern was observed for ceftriaxone concentration prediction, where *K. pneumoniae* overwhelmingly dominated the model (Gain = 0.519), accounting for more than half of the total variable importance. This variable also exhibited exceptionally high Cover (0.508) and Frequency (0.263), indicating both widespread occurrence and repeated selection throughout the boosted trees. The second most influential predictor was *Acinetobacter baumannii* (Gain = 0.321), followed by *Serratia marcescens* (0.150), whereas all remaining variables contributed minimally. For sulfamethoxazole concentration prediction, Site R again represented the principal explanatory variable (Gain = 0.329), followed by *K. pneumoniae* (0.149), *A. veronii* (0.105), *Citrobacter freundii* (0.101), and Time October (0.096). The relatively balanced distribution of Gain values among these predictors indicates that sulfamethoxazole concentrations were influenced by both environmental characteristics and the abundance of multiple bacterial taxa. For azithromycin concentration prediction, the most influential predictor was *E. coli* (Gain = 0.277), followed by Site R (0.230), Time October (0.153), and *K. pneumoniae* (0.055). The predominance of *E. coli*, together with the relatively high importance of fecal indicator organisms, suggests that azithromycin occurrence was more closely associated with indicators of wastewater contamination than with environmental descriptors alone. Across all antibiotics, three consistent patterns emerged. First, Site R was among the most influential predictors for trimethoprim, ciprofloxacin, sulfamethoxazole, and azithromycin, highlighting the importance of spatial heterogeneity in determining antibiotic concentrations. Second, Time October repeatedly ranked among the leading predictors, particularly for amoxicillin and azithromycin, indicating a measurable seasonal effect. Third, several opportunistic and clinically relevant bacterial taxa, especially *K. pneumoniae*, *E. coli*, *C. freundii*, and *A. veronii*, consistently exhibited high Gain values, supporting their strong association with antibiotic occurrence. Conversely, taxa such as *Pseudomonas putida*, *Pseudomonas fulva*, *Pseudomonas monteilii*, *Pseudomonas* sp., and *Ochrobactrum intermedium* displayed extremely low Gain values (typically < 10^−3^), despite occasionally exhibiting moderate Cover or Frequency, indicating that their inclusion contributed little to predictive accuracy.

## 3. Discussion

### 3.1. Occurrence of Antibiotics in Wastewater and Surface-Water Samples

The present study demonstrates that antibiotic occurrence in wastewater and surface waters in the Novi Sad metropolitan area is characterized by pronounced compound-specific differences and that these differences can be partially captured using environmental and physicochemical indicators. All seven target antibiotics were detected in at least one sample, but their frequencies, concentration ranges, seasonal patterns, and distribution between wastewater and surface water varied substantially. Azithromycin was the most frequently detected antibiotic, followed by sulfamethoxazole, trimethoprim, and ciprofloxacin, whereas amoxicillin, ceftriaxone, and doxycycline occurred less frequently. The broader antibiotic profiles and markedly higher concentrations found in wastewater confirm its role as the principal immediate source of antibiotic residues entering the local aquatic environment. At the same time, the detection of several antibiotics in surface water demonstrates that dilution, transformation, and sorption processes do not completely eliminate these compounds after their release. The predominance of antibiotics in wastewater relative to surface water is consistent with the established understanding that municipal wastewater receives residues excreted after human antibiotic consumption, together with compounds originating from hospitals, households, improper pharmaceutical disposal, and potentially veterinary or industrial sources [19,20,21]. The observed concentrations and detection patterns are also broadly consistent with previous investigations of pharmaceutical contamination in the Serbian section of the Danube. Radović et al. detected pharmaceuticals in surface and groundwater samples collected along the Danube and its tributaries in Serbia, including azithromycin at concentrations of 25–140 ng/L [22]. Subsequent studies conducted in Serbia and the Budapest region have likewise confirmed that the Danube receives complex mixtures of pharmaceuticals and that compound-specific persistence governs their transport into connected aquatic compartments [23]. The greater frequency of azithromycin in the present surface-water samples than in the earlier Serbian survey may reflect differences in sampling locations, analytical sensitivity, antibiotic consumption, wastewater inputs, hydrological conditions, and the timing of the sampling campaigns. Consequently, comparisons among monitoring studies should account not only for concentration ranges, but also for differences in detection limits, sample matrices, flow conditions, and sampling design.

The different occurrence profiles of the seven antibiotics can be explained partly by their contrasting environmental properties. Sulfamethoxazole and trimethoprim are relatively mobile in the aqueous phase and are therefore frequently detected in wastewater-impacted rivers. In contrast, fluoroquinolones such as ciprofloxacin have a strong affinity for suspended matter, organic particles, and sediments because of their ionic character and high sorption potential. Tetracyclines, including doxycycline, likewise show substantial sorption and complexation with mineral surfaces and polyvalent cations [20,24]. These properties provide a plausible explanation for the absence of doxycycline from surface-water samples despite its detection in wastewater. The compound may have been removed from the dissolved phase through sorption, transformation, or dilution below the analytical detection limit. Ceftriaxone and amoxicillin are β-lactam antibiotics and are generally less environmentally persistent because the β-lactam ring is susceptible to hydrolysis and other transformation reactions. Their low detection frequencies, particularly the absence of ceftriaxone from surface water, should therefore not be interpreted as evidence that these antibiotics were not discharged. Rather, it may indicate rapid transformation, episodic inputs, matrix-dependent analytical losses, or concentrations below the method detection limit. The seasonal patterns were highly compound-specific. Trimethoprim and sulfamethoxazole were more frequently detected in surface water during autumn, whereas ciprofloxacin was detected only during summer. Azithromycin occurred with the same frequency in both seasons. Amoxicillin and ceftriaxone were detected in wastewater only during autumn, while doxycycline was detected only during summer. Similar compound-dependent seasonality has been reported in river monitoring studies, with changes attributed to antibiotic-consumption patterns, river discharge, precipitation, temperature, sunlight intensity, wastewater dilution, surface runoff, and degradation kinetics [20,21,23]. Lower river discharge can increase concentrations by reducing dilution, whereas rainfall may either dilute wastewater-derived contaminants or increase inputs through combined sewer overflows and agricultural runoff. Higher summer temperatures and stronger solar radiation may accelerate biodegradation and photolysis but can also coincide with lower flow and therefore greater contaminant concentration. Because only two sampling periods were included and each matrix–season subgroup contained nine samples, the present seasonal results should be interpreted as preliminary patterns. Year-round monitoring over multiple years would be necessary to separate seasonal effects from short-term hydrological events and temporal changes in antibiotic consumption [25,26,27].

The frequent occurrence of azithromycin, sulfamethoxazole, trimethoprim, and ciprofloxacin is environmentally relevant because these compounds have been repeatedly associated with antimicrobial-resistance selection in wastewater and receiving waters. Predicted no-effect concentrations for resistance have been proposed as screening thresholds below which selection is considered less likely [28]. Experimental studies have nevertheless demonstrated that resistance determinants can be enriched at antibiotic concentrations substantially below the clinical minimum inhibitory concentrations. Lundström et al. reported the selection of tetracycline-resistance genes in complex aquatic biofilms at concentrations as low as 1 µg/L [29]. The SELECT assay subsequently identified particularly low predicted no-effect concentrations for resistance to ciprofloxacin and indicated that azithromycin and trimethoprim can also present environmental selection risks [30]. Analysis of measured concentrations in wastewater and receiving waters in the United Kingdom further identified ciprofloxacin as a prominent driver of potential resistance selection [31]. Therefore, even though a formal resistance-risk quotient was not calculated in the present study, the occurrence of these antibiotics in wastewater and surface water supports the need to integrate chemical monitoring with measurements of resistant bacteria, antibiotic-resistance genes, mobile genetic elements, and microbial-community composition.

### 3.2. Predictive Performance Using Environmental and Physicochemical Variables

The machine-learning results indicate that the relationships between antibiotic concentrations and the measured environmental conditions were not adequately represented by any single standalone algorithm. The ANN, RF, SVM, and XGBoost models generally showed low to moderate predictive performance, with maximum cross-validated R^2^ values of 0.360 or lower. This finding is not unexpected because antibiotic occurrence is controlled by multiple interacting processes, including source intensity, dilution, sorption, degradation, ionization, hydrological transport, particle dynamics, and microbial transformations. Moreover, the measured physicochemical variables act mainly as indirect proxies for these processes rather than as direct measures of antibiotic emissions. A particular COD, turbidity, or conductivity value may therefore correspond to different antibiotic concentrations depending on wastewater composition, river flow, previous rainfall, treatment efficiency, and the time elapsed between discharge and sampling. The performance of the individual models also illustrates the difficulty of selecting a universally optimal algorithm for environmental pollutant concentration prediction. ANN produced the best standalone R^2^ for trimethoprim, ciprofloxacin, and sulfamethoxazole; RF performed numerically best for amoxicillin; XGBoost was best for ceftriaxone; and SVM was the strongest individual model for azithromycin and doxycycline. These differences suggest that the response surfaces varied among antibiotics and that each algorithm captured a different component of the underlying data structure. ANN can represent complex continuous relationships but is sensitive to network configuration and sample size. SVM can perform well with limited and high-dimensional data but depends strongly on kernel and parameter selection. RF is strong to nonlinearities and interactions, whereas XGBoost can capture sequential residual structures but may overfit sparse datasets if tree depth and boosting iterations are not carefully controlled [32,33].

The STACK-LM approach improved R^2^ relative to the standalone models for all seven antibiotics, indicating that combining base-model predictions contained useful complementary information. However, the improvements remained modest for antibiotics with few detections, particularly amoxicillin, ceftriaxone, and doxycycline. In contrast, STACK-RF achieved the highest numerical performance for every target antibiotic, with R^2^ values ranging from 0.745 for azithromycin to 0.913 for amoxicillin. This result is consistent with the theoretical rationale of stacked generalization, in which a meta-learner combines predictions from structurally different algorithms and reduces model-specific bias when their errors are not perfectly correlated [17]. The favor performance of the nonlinear RF meta-learner relative to the linear meta-model further indicates that the optimal combination of base learners was itself nonlinear and may have varied over the range of predicted concentrations. Comparable improvements from ensemble and stacked approaches have been reported in broader water-quality applications. Park et al. demonstrated that ensemble learning combined with explainable artificial intelligence could predict water-quality variables while identifying the environmental conditions driving the predictions [12]. Stacked regression has also been successfully used for water-quality-index prediction, where combinations of algorithms provided the most favorable numerical predictive performance individual learners and SHAP analysis identified dissolved oxygen, biochemical oxygen demand, conductivity, and pH as influential variables [32]. Most directly relevant to the present work, Jiang et al. showed that ciprofloxacin concentrations could be predicted from conventional water-quality indicators, including COD, dissolved oxygen, water temperature, nitrogen variables, and pH, and reported R^2^ = 0.936 for an optimized generalized regression neural network [10]. The present findings extend this concept from a single antibiotic to seven compounds representing different structural and therapeutic classes. They also indicate that an ensemble can be advantageous when the same predictor set is expected to contain different nonlinear information for different antibiotics [16].

Nevertheless, the very large increase from standalone-model performance to STACK-RF performance requires careful validation. The dataset included only 36 samples, while the predictor matrix contained nine continuous environmental variables and categorical representations of site and sampling time. Under fivefold cross-validation, each test fold therefore contained only approximately seven or eight observations. For amoxicillin, ceftriaxone, and doxycycline, the number of positive detections was particularly low, meaning that the distribution of one or two detected samples among folds could substantially affect the R^2^, RMSE, and MAE. Small analytical datasets are especially sensitive to the selected validation split, model complexity, variable selection, and hyperparameter optimization [33]. Fivefold cross-validation maximizes the use of the available observations, but it does not replace independent validation and cannot introduce environmental variation absent from the original dataset. For stacking, it is particularly important that the meta-learner is trained exclusively on out-of-fold predictions generated without access to the corresponding response observations. Any reuse of fitted base-model predictions during meta-model training can result in information leakage and overly optimistic estimates. In a small dataset, a nested cross-validation structure is preferable, with all preprocessing, dummy-variable encoding, hyperparameter selection, base-model fitting, and meta-learner training repeated independently within each outer training fold [34]. The current STACK-RF results should therefore be described as strong numerical proof-of-concept performance rather than evidence of a deployment-ready predictive model. External validation using samples collected during additional seasons, at new locations, and under different hydrological conditions is needed before the model can be considered transferable to routine Danube monitoring. The interpretation of R^2^ also requires some qualification. In this study, R^2^ was calculated as the squared Pearson correlation between the observed and predicted values. This measure quantifies the strength of linear association but does not fully evaluate agreement or calibration. Predictions may be strongly correlated with observations while remaining systematically biased in magnitude. Correlation-based R^2^ should therefore be interpreted together with RMSE, MAE, SSE, observed-versus-predicted plots, and an assessment of prediction bias [35]. The consistent reduction in RMSE, MAE, and SSE achieved by STACK-RF supports its numerical advantage, but confidence intervals around these metrics would provide a more informative estimate of their stability. The extremely large AARD values should not be interpreted as conventional percentage errors. Percentage-based error measures become undefined or arbitrarily large when the observed values equal or approach zero [36]. This is directly relevant because non-detections were represented by zeros in the analytical dataset. Division by zero or near-zero concentrations produces the very large AARD values reported in Table 2 and prevents meaningful comparison among antibiotics or models. AARD should therefore either be omitted from the principal interpretation or recalculated using a clearly justified treatment of non-detects. Suitable alternatives could include MAE and RMSE on a log-transformed concentration scale, mean absolute scaled error, or a two-stage modeling framework in which the probability of detection is predicted first, and the positive concentrations are subsequently modeled using regression.

The Diebold–Mariano analysis provides an important counterbalance to the descriptive ranking of the models. Before adjustment for multiple testing, several comparisons involving STACK-RF produced nominal *p*-values below 0.05, particularly for trimethoprim, ciprofloxacin, amoxicillin, and sulfamethoxazole. After Holm correction, however, only the SVM–XGBoost and XGBoost–STACK-RF comparisons for amoxicillin concentration remained significant. Thus, although STACK-RF achieved the highest R^2^ and lowest absolute errors for all seven antibiotics, the null hypothesis of equal predictive accuracy could not be rejected for most pairwise comparisons. The appropriate conclusion is therefore that STACK-RF was numerically, but not consistently statistically, superior. The absence of statistical significance does not demonstrate that all models had identical predictive performance. The DM tests may have had limited power because of the small number of predictions and the high variability associated with sparse concentration data. Moreover, the DM test was originally developed to compare forecast-error series [37]. Its application to residuals pooled from randomly generated cross-validation folds should be considered supplementary because the assumptions regarding the dependence structure of forecast losses may not be fully satisfied. Diebold later emphasized that the test evaluates predictive accuracy of competing forecasts rather than establishing general model favorability and warned against overextension of its interpretation [38]. In the present study, the DM analysis appropriately limits overly strong claims based solely on R^2^ rankings. Repeated nested cross-validation, bootstrap confidence intervals for performance differences, or model-comparison procedures specifically adapted to resampling designs would provide stronger evidence in a future expanded dataset. The feature-importance and SHAP results indicate that antibiotic prediction relied primarily on local water-quality and meteorological conditions. COD and pH were the leading predictors for trimethoprim, while turbidity and electrical conductivity dominated ciprofloxacin prediction. Amoxicillin was associated with pH, water temperature, and relative humidity; ceftriaxone was dominated by relative humidity; sulfamethoxazole was linked primarily to turbidity, pH, and COD; azithromycin was associated with BOD_5_, COD, and relative humidity; and doxycycline was dominated by electrical conductivity and water temperature. The heterogeneity of these profiles supports the premise that individual antibiotic classes respond differently to environmental conditions. The importance of COD and BOD_5_ for trimethoprim and azithromycin is environmentally plausible because these variables indicate biodegradable and chemically oxidizable organic loads and can act as proxies for wastewater influence. Elevated organic load may coincide with the release of pharmaceutical residues, nutrients, fecal microorganisms, and other contaminants from the same source. However, COD and BOD_5_ can also influence antibiotic fate indirectly through competition for sorption sites, light attenuation, redox conditions, and microbial activity. Their importance should therefore be understood as a combined source-and-process signal. The strong role of turbidity in ciprofloxacin and sulfamethoxazole prediction may reflect inputs of suspended particles during runoff or wastewater discharge, together with compound-specific interactions with particulate matter. The association is especially plausible for ciprofloxacin because fluoroquinolones readily adsorb to mineral and organic particles. Turbidity may consequently provide information on both contaminant source intensity and partitioning between dissolved and particle-associated phases. For sulfamethoxazole, which is generally more mobile, turbidity may instead act mainly as a marker of runoff, disturbed sediment, or wastewater mixing. Measuring both dissolved and particle-bound antibiotic fractions would be necessary to distinguish these mechanisms. Electrical conductivity was particularly influential for ciprofloxacin and doxycycline. Conductivity reflects the total concentration of dissolved ions and can indicate wastewater mixing, mineral inputs, saline influence, or changes in ionic strength. Ionic composition can affect antibiotic speciation, complexation, and sorption, particularly for amphoteric compounds such as fluoroquinolones and tetracyclines. Water temperature can modify hydrolysis, photochemical reactions, microbial transformation rates, oxygen solubility, and sorption equilibria, providing a plausible basis for its importance in the amoxicillin and doxycycline models. Similarly, pH affects the ionization state of most antibiotics and therefore their aqueous solubility, charge, reactivity, and affinity for suspended matter. Its importance for trimethoprim, amoxicillin, and sulfamethoxazole is consistent with the pH-dependent environmental behavior of these compounds. Relative humidity was identified as an important predictor for amoxicillin, ceftriaxone, and azithromycin, but this relationship requires particularly cautious interpretation.

Relative air humidity was an influential predictor in the XGBoost models for amoxicillin, ceftriaxone, and azithromycin, but its importance should be interpreted as a statistical association within the available dataset. Relative humidity does not directly quantify antibiotic inputs, river dilution, or contaminant transport. It may covary with sampling-campaign conditions or unmeasured meteorological and hydrological processes; however, these potential relationships were not tested in the present study. Its high importance for ceftriaxone warrants particular caution because only three wastewater samples, all collected during autumn, contained detectable concentrations. Moreover, the low predictive performance of the standalone XGBoost model limits the environmental interpretation of its feature rankings. Neither Gain nor SHAP values establish causality, and XGBoost feature importance should not be interpreted as a direct measure of predictor importance in the STACK-RF ensemble. The current predictor set did not include river discharge, antecedent dry periods, wastewater-flow measurements, antibiotic-use data, or heavy-metal concentrations. Their omission limits the ability to distinguish variation in contaminant inputs from dilution, transport, and shared pollution-source effects. Future monitoring should prioritize river discharge and wastewater flows matched to sampling locations and dates, together with antecedent precipitation and dry-period duration. These measurements would help characterize dilution and hydrological transport, and where representative concentration measurements are available, permit the estimation of antibiotic loads. Catchment-relevant human and veterinary antibiotic-use data, with appropriate temporal resolution and consideration of time lags, would provide additional information on potential emission pressures. Heavy metals and other co-selective contaminants should also be considered, particularly when interpreting associations between antibiotic residues and presumptive resistant bacterial taxa. Such contaminants may contribute to the selection or persistence of resistant populations independently of the measured antibiotic concentrations. Their inclusion could therefore help investigate whether microbial predictors reflect shared contamination sources or additional selective pressures. Any improvement in predictive performance or transferability from these additional variables would need to be demonstrated using expanded datasets and independent validation. Additionally, water temperature is related to the time of year, but the two variables represent different aspects of the sampled system. Sampling time distinguished the summer and autumn campaigns, whereas measured water temperature described the thermal conditions at individual sampling events. Temperature may therefore capture variation both between and within campaigns that is not fully represented by a categorical time variable. When predictors contain overlapping information, a model may assign greater importance to one of them; consequently, the comparatively low importance of sampling time does not demonstrate that seasonal influences are negligible. Likewise, high temperature importance does not establish a statistically significant or independent causal temperature effect. Differences between sampling periods may reflect concurrent changes in antibiotic inputs, transport, dilution, and transformation. Human antibiotic-use patterns may alter wastewater inputs, while veterinary treatments, livestock management, manure application, and subsequent runoff may contribute additional temporal variation where these sources are present. Rainfall can increase contaminant delivery through runoff, and where applicable, sewer overflows, but can also increase river discharge and dilute existing contamination. Conversely, low-flow conditions may increase concentrations without an increase in antibiotic mass input. Higher water temperatures can accelerate some hydrolytic and microbial transformation processes, while seasonal changes in solar radiation may independently affect photodegradation. These processes can act in opposing directions, and their combined effects depend on the antibiotic, water matrix, and local conditions.

SHAP analysis adds value by showing how variables contribute to fitted predictions, but SHAP values must not be interpreted as causal effects [15]. Environmental predictors are frequently correlated, and attribution can be distributed among related variables such as COD, BOD_5_, turbidity, conductivity, temperature, and humidity. A high SHAP importance therefore means that a variable was useful to the fitted model in the context of the other available predictors; it does not prove that experimentally altering the variable would alter the antibiotic concentration. Likewise, the absolute IncNodePurity values from separate STACK-RF models should not be compared directly among antibiotics because the scale and variance of the response strongly affect impurity reductions. Random forest importance measures can also be biased by predictor scale, number of possible split points, and correlations among predictors [39,40]. Within-model rankings are consequently more informative than comparisons of absolute IncNodePurity values across antibiotic-specific models.

### 3.3. Predictive Performance Using Presumptive Resistant Bacterial Taxa Detected in Wastewater and Surface Water Samples

The second part of this study demonstrates that predicting antibiotic concentrations from the occurrence profiles of culture-based presumptive resistant bacterial taxa is a complex nonlinear problem that cannot be adequately resolved by conventional standalone machine-learning algorithms. Although ANNs, RFs, SVMs, and XGBoosts have been successfully applied in numerous environmental prediction studies, all four individual algorithms exhibited poor predictive performance in the present dataset, explaining less than 11% of the variance for all investigated antibiotics. Such results suggest that the relationship between occurrence profiles of culture-based presumptive resistant bacterial taxa and antibiotic occurrence is characterized by complex interactions, nonlinear dependencies, and potentially high-dimensional feature spaces that cannot be sufficiently represented by a single learning algorithm. In contrast, the substantial improvement achieved by the stacking ensembles, particularly STACK-RF, demonstrates that combining complementary prediction strategies enables a more effective extraction of information from heterogeneous microbial datasets. The favorability of the STACK-RF model was remarkably consistent across all investigated antibiotics [18]. Coefficients of determination increased from values close to zero for the standalone models to 0.819–0.945 for the ensemble, accompanied by substantial reductions in RMSE, MAE, AARD, and SSE. Such improvements indicate that the ensemble approach successfully integrated the strengths of different base learners while compensating for their individual weaknesses. Ensemble learning generally reduces both variance and prediction bias through the aggregation of multiple models, leading to greater accuracy when relationships between predictors and response variables are highly nonlinear [41]. The observed performance therefore suggests that antibiotic occurrence is governed by multiple interacting biological and environmental processes whose combined effects are more effectively captured through stacked learning than through any individual algorithm.

Despite the pronounced numerical favorability of the STACK-RF model, the Diebold–Mariano analysis showed that relatively few pairwise differences remained statistically significant after Holm correction. Only the comparisons between STACK-RF and ANN for ciprofloxacin and between STACK-RF and XGB for sulfamethoxazole concentration prediction retained statistical significance. This apparent discrepancy between predictive metrics and statistical testing is not unexpected. The Diebold–Mariano test evaluates differences in forecast errors while accounting for their variability, whereas regression metrics summarize overall predictive performance. Consequently, substantial improvements in R^2^ and prediction error may not necessarily translate into statistical significance when prediction errors exhibit considerable variability or when the number of observations is limited. Moreover, Holm adjustment represents a conservative procedure for controlling the family-wise error rate and consequently reduces statistical power. Therefore, the absence of statistical significance for most comparisons should not be interpreted as evidence of equivalent predictive performance. The feature importance analysis further supports the hypothesis that antibiotic occurrence is jointly determined by the environmental characteristics and occurrence profiles of culture-based presumptive resistant bacterial taxa [42]. Among the environmental descriptors, sampling site consistently emerged as one of the most influential variables for the trimethoprim, ciprofloxacin, sulfamethoxazole, and azithromycin concentrations [43]. This finding indicates pronounced spatial heterogeneity in antibiotic contamination, which is consistent with differences in wastewater inputs, surrounding land use, hydrological conditions, and local anthropogenic activities. Spatial variability has repeatedly been identified as one of the principal determinants of antibiotic occurrence in aquatic environments [44], reflecting variations in contamination sources, dilution processes, and environmental transport. Temporal variation also contributed substantially to model predictions. The importance of the October sampling period, particularly for the amoxicillin and azithromycin concentrations, suggests that seasonal conditions influence antibiotic concentrations, either through changes in antibiotic consumption patterns, wastewater discharge, river flow, or environmental degradation processes. Seasonal fluctuations in precipitation, temperature, and microbial activity are known to modify both antibiotic persistence and bacterial community structure [45], providing a plausible explanation for the observed temporal importance.

Among the microbial predictors, *Klebsiella pneumoniae*, *Escherichia coli*, *Citrobacter freundii*, *Aeromonas veronii*, *Acinetobacter baumannii*, and *Serratia marcescens* consistently ranked among the most influential taxa. Most of these organisms are well-recognized indicators of wastewater contamination and are frequently associated with the dissemination of antibiotic resistance genes. Their strong importance therefore likely reflects their role as ecological indicators of anthropogenic pollution. In particular, *E. coli* is widely used as fecal indicator bacteria, whereas *K. pneumoniae*, *A. baumannii*, and *Serratia marcescens* are opportunistic pathogens commonly encountered in hospitals and municipal wastewater [46]. Their repeated identification as dominant predictors suggests that the occurrence profiles of culture-based presumptive resistant bacterial taxa contain valuable information regarding the intensity and origin of antibiotic contamination. The dominant predictor differed among antibiotics, indicating that different compounds may be associated with distinct ecological signatures [47]. Spatial location primarily determined the trimethoprim, ciprofloxacin, and sulfamethoxazole concentration predictions, whereas the amoxicillin and ceftriaxone concentrations were predominantly associated with specific bacterial taxa, especially *K. pneumoniae*. Azithromycin concentration, on the other hand, was most strongly associated with *E. coli*. These differences likely reflect the distinct physicochemical properties, environmental persistence, degradation pathways, and usage patterns of individual antibiotics. Consequently, microbial communities appear to respond differently to each compound, reinforcing the need for antibiotic-specific predictive models rather than a single generalized approach [48,49]. An equally important observation is that many bacterial taxa exhibited extremely low Gain values despite appearing relatively frequently within the decision trees. Species belonging to *Pseudomonas* and *Ochrobactrum*, for example, occasionally showed moderate Cover or Frequency but contributed negligibly to prediction accuracy. This finding illustrates that variables may participate repeatedly in tree construction without substantially improving model performance and emphasizes the importance of evaluating feature contribution using Gain rather than selection frequency alone. The results also indicate that only a relatively small subset of bacterial taxa contains meaningful predictive information, whereas the remaining variables contribute primarily noise. The combination of predictive modeling and SHAP-based interpretation therefore provides complementary insights into antibiotic occurrence [50,51]. While the stacking models maximize predictive accuracy, the XGBoost interpretation identifies biologically meaningful predictors that may serve as potential indicators of antibiotic contamination. Such interpretable machine-learning approaches are particularly valuable in environmental microbiology because they simultaneously improve prediction and enhance the understanding of ecological processes governing contaminant distribution. Several limitations should nevertheless be acknowledged. The limited statistical significance observed in the Diebold–Mariano analysis suggests that a larger dataset covering additional sampling locations, seasons, and environmental conditions would improve statistical power and permit more robust comparison among competing algorithms. Furthermore, occurrence profiles of culture-based presumptive resistant bacterial taxa represent only one component of the environmental system controlling antibiotic occurrence. Incorporating complementary physicochemical variables, hydrological parameters, wastewater discharge characteristics, antibiotic resistance genes, and environmental metadata would likely improve both predictive accuracy and model interpretability. External validation using independent datasets from different geographical regions would also be necessary to assess the generalizability of the developed models.

## 4. Materials and Methods

### 4.1. Sampling

Sample collection was performed within the TRACE project (Grant No. 7042, WP4) in the metropolitan area of Novi Sad, Serbia. Surface-water and wastewater samples were collected at official sampling locations used by the public utility company “Vodovod i kanalizacija” Novi Sad. The study followed the company’s established sampling protocols and operational sequence of site visits. Two sampling campaigns were conducted during 1–15 July 2024 and 1–15 October 2024. A total of 36 samples were analyzed, comprising 18 wastewater and 18 surface-water samples, with nine samples from each matrix collected during each campaign. These dates define the campaign windows and do not imply continuous daily sampling. Sample collection followed the company’s established procedures within each campaign.

The sampling procedure was conducted by the public utility company “Vodovod i kanalizacija” Novi Sad in compliance with standard practices for collecting environmental water samples. Sterile sampling containers prepared in advance by the project research team were used to minimize the risk of external contamination. Immediately after collection, the samples were handed over to the project team and transported under controlled conditions to preserve their original microbiological and chemical characteristics. A continuous cold chain was maintained at 4 ± 1 °C using portable cooling equipment (Zonkia, Hefei City, China), following established requirements for environmental microbiological and chemical investigations. All collected samples were subsequently delivered to the laboratories of the Faculty of Medicine, University of Belgrade, where further microbiological characterization was performed.

### 4.2. Environmental Parameters and Physicochemical Analysis

Environmental and physicochemical variables were documented at every sampling site and subsequently included as environmental predictors in the statistical and machine learning models. The selected variables represented both prevailing meteorological conditions and water-quality properties potentially associated with the occurrence of antibiotic resistance and the presence of antibiotic residues. Air temperature (°C), relative humidity (%), and atmospheric pressure (hPa) at the time of sampling were retrieved from validated online meteorological databases. Water-quality parameters were determined through a combination of field and laboratory measurements. The analyzed physicochemical characteristics included water temperature (°C), turbidity (NTU), pH, and electrical conductivity (µS/cm). Indicators of organic contamination were also assessed, including chemical oxygen demand, expressed as COD in mg O_2_/L and determined by the dichromate method, and five-day biochemical oxygen demand, expressed as BOD_5_ in mg O_2_/L. All measurements were carried out by the accredited laboratory of the public utility company “Vodovod i kanalizacija” Novi Sad using standardized analytical procedures based on the Standard Methods for the Examination of Water and Wastewater (SMEWW) and relevant EPA protocols. Water temperature and electrical conductivity were measured according to SMEWW methods 2550B and 2510B, respectively, while pH was determined using SMEWW method 4500-H^+^. Turbidity was measured in accordance with EPA method 180.1. COD and BOD_5_ were quantified using validated internal procedures harmonized with applicable regulatory requirements. The resulting environmental and water-quality dataset was used as a set of input variables in subsequent statistical and machine learning analyses to investigate the contribution of these factors to variations in antibiotic concentrations.

### 4.3. Sample Processing and Antibiotic Quantification

Selected antibiotics (sulfamethoxazole, trimethoprim, azithromycin, doxycycline, amoxicillin, ceftriaxone, and ciprofloxacin) were extracted from surface-water and wastewater samples using solid-phase extraction (SPE). Before extraction, the sample pH was adjusted to 3.0. Surface-water samples (200 mL) and wastewater samples (100 mL) were loaded onto Oasis HLB cartridges (200 mg/6 mL; Waters), previously conditioned with 5 mL of methanol, 5 mL of deionized water, and 5 mL of deionized water adjusted to pH 3.0. After sample loading, the cartridges were dried under vacuum for 10 min, and the analytes were eluted with 15 mL of methanol. The eluates were evaporated to dryness under a nitrogen stream at 30 °C, reconstituted in 1 mL of methanol, filtered through a 0.45 µm PVDF membrane, and subjected to HPLC-MS/MS analysis. Chromatographic separation was performed using a DionexUltiMate 3000 HPLC system, Sunnyvale, CA, USA, equipped with a Zorbax Eclipse XDB-C18 column (4.6 × 75 mm, 3.5 µm) and a matching guard column (4.6 × 12.5 mm, 5 µm). The mobile phase consisted of methanol, deionized water, and 10% (*v*/*v*) formic acid. Two gradient programs were applied. For sulfamethoxazole, trimethoprim, azithromycin, doxycycline, amoxicillin, ceftriaxone, and ciprofloxacin, the methanol content increased from 30% to 60% over 12 min and then to 99% at 15 min, followed by column washing and re-equilibration, giving a total run time of 40 min. Mass-spectrometric detection was performed using an LTQ XL linear ion-trap mass spectrometer, San Jose, CA, US, operated in positive electrospray ionization mode. The capillary temperature, nitrogen flow, and source voltage were set at 220 °C, 22 arbitrary units, and 5.0 kV, respectively. Quantification was based on the following precursor-to-product ion transitions: *m*/*z* 254.2 → 188.1 for sulfamethoxazole, 291.2 → 230.2 for trimethoprim, 375.5 → 591.6 for azithromycin, 445.3 → 428.3 for doxycycline, 398.2 → 381.2 for amoxicillin, 555.1 → 396.1 for ceftriaxone, 332.3 → 314.2 for ciprofloxacin. Instrumental data were processed using Xcalibur v.2.2. Antibiotic concentrations in environmental samples were determined using the standard-addition method to account for matrix effects and incomplete analyte recovery. Method detection limits were established as the lowest concentrations producing a signal-to-noise ratio of 3.

### 4.4. Machine Learning Modeling and Model Evaluation

Machine learning (ML) models were developed to predict the concentrations of seven antibiotics, namely trimethoprim, ciprofloxacin, amoxicillin, ceftriaxone, sulfamethoxazole, azithromycin, and doxycycline, using environmental and physicochemical parameters as predictors. The input variables included two categorical variables (sampling site and sampling time) and nine continuous physicochemical variables: air temperature, relative air humidity, atmospheric pressure, water temperature, turbidity, pH, electrical conductivity, COD, and BOD_5_. In addition, the microbiological dataset was used as input factors in a second set of predicting models for antibiotic concentration, including the species *Acinetobacter baumannii*, *Acinetobacter courvalinii*, *Aeromonas veronii*, *Citrobacter braakii*, *Citrobacter freundii*, *Enterobacter hormaechei*, *Escherichia coli*, *Klebsiella pneumoniae*, *Klebsiella variicola*, *Ochrobactrum intermedium*, *Pseudomonas fulva*, *Pseudomonas guariconensis*, *Pseudomonas monteilii*, *Pseudomonas putida*, *Pseudomonas* sp., *Serratia marcescens*, and *Stenotrophomonas maltophilia*. All data on the detected and identified bacteria are reported in the study by Jovićević et al. [18].

The dataset was imported into R software (version 4.4.3, R Foundation for Statistical Computing, Vienna, Austria). Empty cells were treated as missing values and subsequently removed using complete-case analysis. Continuous predictor variables were converted to numeric format, whereas the categorical variables Site and Time were encoded as factors. To enable the use of machine learning algorithms requiring numerical input, categorical variables were transformed into dummy variables using a design matrix generated by the model. Five machine learning algorithms were evaluated: artificial neural networks (ANNs), random forest (RF), support vector machines (SVMs), Extreme Gradient Boosting (XGBoost), and two ensemble stacking approaches (stacked linear model, STACK-LM, and stacked random forest, STACK-RF). Model development and evaluation were performed separately for each antibiotic. Model performance was assessed using fivefold cross-validation [52]. The dataset was randomly divided into five approximately equal subsets. During each iteration, four subsets were used for model training, and the remaining subset was used for testing. This procedure was repeated until each subset had served once as the testing set. Predictions obtained from all testing folds were combined to generate out-of-fold predictions for the complete dataset. The random forest model was implemented using 500 decision trees. The support vector machine model employed a radial basis function kernel. Artificial neural networks were developed using a multilayer perceptron architecture with a single hidden layer consisting of eight neurons, linear output activation, and a maximum of 1000 training iterations. The XGBoost model was implemented using gradient-boosted decision trees with a learning rate (*η*) of 0.05, maximum tree depth of 6, subsampling ratio of 0.80, column sampling ratio of 0.80, and 200 boosting rounds. The objective function was defined as squared-error regression, and model performance during training was monitored using the root mean square error (RMSE). To improve predictive performance, ensemble stacking models were developed. Predictions generated by the ANN, RF, SVM, and XGB models were used as input variables for the meta-models. Two stacking approaches were evaluated: (a) a multiple linear regression model (STACK-LM) and (b) a random forest meta-model containing 500 trees (STACK-RF). The stacking models combined the predictions of the base learners to generate final ensemble predictions. Model performance was evaluated using the coefficient of determination (R^2^), root mean square error (RMSE), mean absolute error (MAE), average absolute relative deviation (AARD), and sum of squared errors (SSE). The coefficient of determination was calculated as the squared Pearson correlation coefficient between the observed and predicted values. Lower RMSE, MAE, AARD, and SSE values, together with higher R^2^ values, indicated favorable predictive performance. The relative importance of predictor variables was investigated using model-specific explainability methods. Feature importance scores were extracted from the XGBoost and STACK-RF models. In addition, SHapley Additive exPlanations (SHAP) analysis was performed using the final XGBoost models fitted to the complete dataset. Finally, pairwise statistical comparisons among the six predictive models (ANN, RF, SVM, XGB, STACK-LM, and STACK-RF) were performed using the Diebold–Mariano (DM) test. The DM test evaluates whether the forecast errors produced by two competing models differ significantly. Pairwise comparisons were conducted using one-step-ahead forecast errors, and *p*-values below 0.05 were considered indicative of statistically significant differences in predictive accuracy.

The developed machine-learning models have potential to support the environmental monitoring of antibiotics by providing rapid estimates of antibiotic concentrations from routinely measured environmental, physicochemical, and microbiological parameters. Such predictions could assist in identifying periods or locations with potentially elevated contamination and could be used to support the prioritization of sampling and laboratory analyses. The models are intended to complement, rather than replace, conventional analytical measurements. Future monitoring should include year-round, multi-year sampling with broader spatial coverage and independent validation datasets from additional aquatic systems. Incorporation of hydrological characteristics, wastewater-related parameters, pharmaceutical usage information, and additional microbiological indicators could further improve model robustness and environmental applicability. With sufficiently large datasets, future work could also employ detection-aware or two-stage modeling approaches to distinguish the probability of antibiotic occurrence from the concentration levels. These developments could contribute to more efficient, data-driven antibiotic surveillance and provide an initial basis for integrating machine learning into environmental monitoring frameworks.

## 5. Conclusions

This study provides a proof-of-concept framework for predicting antibiotic residues in wastewater and surface water associated with the Danube River within the territory of the city of Novi Sad by integrating chemical monitoring, environmental measurements, microbiological indicators, stacked ensemble learning, and explainable artificial intelligence. All seven target antibiotics were detected in at least one of the 36 samples, but their occurrence was strongly compound- and matrix-specific. Wastewater generally contained broader antibiotic profiles and higher concentrations than surface water, supporting its role as an important immediate source of antibiotic residues entering the local aquatic environment. Azithromycin was the most frequently detected compound, followed by sulfamethoxazole, trimethoprim, and ciprofloxacin, whereas amoxicillin, ceftriaxone, and doxycycline occurred less frequently. A major strength of the study was the parallel evaluation of two complementary predictor domains: environmental and physicochemical parameters, and presumptive resistant bacterial taxa. The comparison of ANN, RF, SVM, XGBoost, STACK-LM, and STACK-RF models using fivefold cross-validation and multiple performance metrics showed that no single standalone algorithm adequately captured the complex relationships governing antibiotic occurrence. In contrast, STACK-RF consistently produced the highest numerical accuracy and the lowest absolute prediction errors. Its R^2^ values ranged from 0.745 to 0.913 for models based on environmental and physicochemical variables and from 0.819 to 0.945 for the six antibiotics with available microbial-taxa model results. These findings indicate that combining structurally different base learners can capture complementary nonlinear patterns that remain poorly represented by individual algorithms. The explainability analyses further demonstrated that predictor relevance was antibiotic-specific. The COD, BOD_5_, pH, turbidity, electrical conductivity, water temperature, and relative air humidity were among the most influential environmental variables, whereas *Klebsiella pneumoniae*, *Escherichia coli*, *Citrobacter freundii*, and *Aeromonas veronii* repeatedly emerged as informative microbial predictors. These taxa should be interpreted as potential indicators of wastewater influence, shared contamination sources, or ecological conditions associated with antibiotic occurrence rather than as direct determinants of antibiotic concentrations. Accordingly, the Gain and SHAP results are most appropriately viewed as hypothesis-generating evidence rather than confirmation of causal environmental or microbiological mechanisms.

Several limitations restrict the generalizability of the present findings. The dataset comprised only 36 samples collected during two seasonal campaigns within one metropolitan area, limiting statistical power and representation of hydrological and temporal variability. Sparse detections and zero or near-zero concentrations also affected the stability and interpretation of percentage-based errors, particularly AARD. Although fivefold cross-validation reduced dependence on a single train–test split, it cannot replace validation with independent spatial and temporal datasets. In addition, most apparent advantages of STACK-RF were not statistically significant after Holm correction, indicating that its favorability was primarily numerical rather than consistently statistically confirmed. The application of the Diebold–Mariano test to pooled cross-validation residuals should also be considered supplementary because the dependence assumptions of conventional forecast-error testing may not be fully satisfied. Further development should therefore include larger multi-season and multi-site datasets, repeated or nested cross-validation, bootstrap confidence intervals, and external validation in other sections of the Danube and geographically distinct river systems.

Explainable stacked ensemble learning identified nonlinear relationships between environmental conditions and antibiotic occurrence in surface waters and wastewater-impacted areas associated with the Danube River near Novi Sad, Serbia. The results demonstrate the potential of ensemble machine learning for antibiotic-concentration prediction from routinely measurable environmental and water-quality variables. However, the limited number of observations and coverage of only two seasonal campaigns constrain the assessment of long-term seasonal and interannual variability. Extended year-round, multi-year monitoring is therefore required to establish the temporal generalizability of the developed models and to distinguish recurrent seasonal patterns from short-term hydrological and environmental fluctuations.

Future models should integrate river discharge and flow conditions, wastewater-treatment and discharge characteristics, sediment properties, antibiotic-use data, resistance genes, mobile genetic elements, heavy metals, and other co-selective contaminants. A two-stage modeling strategy that first predicts antibiotic detection and subsequently models positive concentrations may also improve performance for sparsely detected compounds. Despite these limitations, the study demonstrates that explainable stacked ensemble learning is a promising complementary tool for identifying nonlinear and antibiotic-specific associations within complex aquatic datasets. With broader validation, such models could support the prioritization of sampling locations and variables, guide hypothesis-driven monitoring, and complement—but not replace—targeted chemical analysis in environmental and One Health surveillance.

## Figures and Tables

**Table 1:** Occurrence and distribution of antibiotic concentrations by sample type and season.

Antibiotic	Sample Type	Detected	Season		Median (Log ng/L)	Range (Log ng/L)
			Summer	Autumn		
Trimethoprim	Wastewater	12/18 (66.7%)	6/9 (66.7%)	6/9 (66.7%)	2.392	1.155–2.891
	Surface water	7/18 (38.9%)	0/9 (0.0%)	7/9 (77.8%)	0.851	0.792–1.017
Ciprofloxacin	Wastewater	14/18 (77.8%)	7/9 (77.8%)	7/9 (77.8%)	2.918	1.497–3.404
	Surface water	5/18 (27.8%)	5/9 (55.6%)	0/9 (0.0%)	1.804	1.553–1.963
Amoxicillin	Wastewater	4/18 (22.2%)	0/9 (0.0%)	4/9 (44.4%)	1.919	1.768–1.940
	Surface water	2/18 (11.1%)	1/9 (11.1%)	1/9 (11.1%)	1.401	1.320–1.470
Ceftriaxone	Wastewater	3/18 (16.7%)	0/9 (0.0%)	3/9 (33.3%)	2.265	1.563–2.548
	Surface water	0/18 (0.0%)	0/9 (0.0%)	0/9 (0.0%)	nd	nd
Sulfamethoxazole	Wastewater	11/18 (61.1%)	5/9 (55.6%)	6/9 (66.7%)	2.760	1.870–3.068
	Surface water	10/18 (55.6%)	3/9 (33.3%)	7/9 (77.8%)	0.484	0.398–0.580
Azithromycin	Wastewater	13/18 (72.2%)	7/9 (77.8%)	6/9 (66.7%)	1.382	0.415–2.436
	Surface water	14/18 (77.8%)	7/9 (77.8%)	7/9 (77.8%)	0.190	0.041–0.756
Doxycycline	Wastewater	2/18 (11.1%)	2/9 (22.2%)	0/9 (0.0%)	2.080	2.034–2.121
	Surface water	0/18 (0.0%)	0/9 (0.0%)	0/9 (0.0%)	nd	nd

nd—non-detection.

**Table 2:** Complete performance metrics for environmental and physicochemical predictor models.

Antibiotic	Model	R^2^	RMSE	MAE	AARD	SSE
Trimethoprim	ANN	0.181	171.525	120.070	3.8 × 10^12^	1.1 × 10^6^
	RF	0.124	186.010	112.275	3.5 × 10^12^	1.2 × 10^6^
	SVM	0.051	191.146	114.672	2.9 × 10^12^	1.3 × 10^6^
	XGB	0.059	207.100	117.481	3.1 × 10^12^	1.5 × 10^6^
	STACK-LM	0.341	153.662	109.180	3.6 × 10^12^	8.5 × 10^5^
	STACK-RF	0.787	100.640	64.259	2.2 × 10^12^	3.6 × 10^5^
Ciprofloxacin	ANN	0.360	526.598	315.222	9.7 × 10^12^	1.0 × 10^7^
	RF	0.147	620.161	380.848	9.7 × 10^12^	1.4 × 10^7^
	SVM	0.030	674.635	387.561	9.7 × 10^12^	1.6 × 10^7^
	XGB	0.105	653.185	379.024	8.0 × 10^12^	1.5 × 10^7^
	STACK-LM	0.543	444.839	302.491	1.0 × 10^13^	7.1 × 10^6^
	STACK-RF	0.822	313.435	186.301	6.1 × 10^12^	3.5 × 10^6^
Amoxicillin	ANN	0.004	28.432	18.975	1.0 × 10^12^	2.9 × 10^6^
	RF	0.046	28.479	19.334	1.0 × 10^12^	2.9 × 10^4^
	SVM	0.001	26.141	13.295	3.9 × 10^11^	2.5 × 10^4^
	XGB	0.041	32.061	21.390	1.2 × 10^12^	3.7 × 10^4^
	STACK-LM	0.154	22.965	15.912	9.0 × 10^11^	1.9 × 10^4^
	STACK-RF	0.913	12.348	8.218	4.3 × 10^11^	5.5 × 10^3^
Ceftriaxone	ANN	0.000	86.919	33.051	1.8 × 10^12^	2.7 × 10^5^
	RF	0.022	64.642	26.121	1.3 × 10^12^	1.5 × 10^5^
	SVM	0.020	65.965	21.228	6.2 × 10^11^	1.6 × 10^5^
	XGB	0.064	69.235	23.042	1.2 × 10^12^	1.7 × 10^5^
	STACK-LM	0.095	61.568	28.768	1.5 × 10^12^	1.4 × 10^5^
	STACK-RF	0.880	35.565	14.045	6.4 × 10^11^	4.6 × 10^4^
Sulfamethoxazole	ANN	0.203	282.981	187.186	6.1 × 10^12^	2.9 × 10^6^
	RF	0.071	319.627	198.145	7.2 × 10^12^	3.7 × 10^6^
	SVM	0.032	322.607	194.216	5.2 × 10^12^	3.7 × 10^6^
	XGB	0.005	367.560	219.626	6.6 × 10^12^	4.9 × 10^6^
	STACK-LM	0.270	269.114	167.925	5.0 × 10^12^	2.6 × 10^6^
	STACK-RF	0.827	155.648	98.330	3.7 × 10^12^	8.7 × 10^5^
Azithromycin	ANN	0.067	51.539	28.320	7.0 × 10^11^	9.6 × 10^4^
	RF	0.084	50.322	25.187	5.2 × 10^11^	9.1 × 10^4^
	SVM	0.232	45.222	18.821	3.7 × 10^11^	7.4 × 10^4^
	XGB	0.063	53.130	24.052	5.6 × 10^11^	1.0 × 10^5^
	STACK-LM	0.244	42.410	20.616	6.5 × 10^11^	6.5 × 10^4^
	STACK-RF	0.745	29.396	12.795	2.7 × 10^11^	3.1 × 10^4^
Doxycycline	ANN	0.000	28.890	12.032	5.7 × 10^11^	3.0 × 10^4^
	RF	0.002	29.480	14.435	8.2 × 10^11^	3.1 × 10^4^
	SVM	0.056	28.457	9.131	2.5 × 10^11^	2.9 × 10^4^
	XGB	0.006	31.850	13.094	6.5 × 10^11^	3.7 × 10^4^
	STACK-LM	0.066	26.745	13.344	7.5 × 10^11^	2.6 × 10^4^
	STACK-RF	0.837	15.534	6.490	3.2 × 10^11^	8.7 × 10^3^

**Table 3:** Diebold–Mariano pairwise comparisons of the environmental and physicochemical predictor models.

	ANN			RF			SVM			XGB			STACK-LM		
Trimethoprim	DM	Statistics	Holm *p*	DM	Statistics	Holm *p*	DM	Statistics	Holm *p*	DM	Statistics	Holm *p*	DM	Statistics	Holm *p*
RF	−1.047	0.302	0.862												
SVM	−1.353	0.185	0.862	−0.410	0.684	0.862									
XGB	−1.812	0.079	0.707	−1.716	0.095	0.760	−1.135	0.264	0.862						
STACK-LM	1.393	0.172	0.862	1.685	0.101	0.760	1.565	0.126	0.760	1.873	0.069	0.695			
STACK-RF	2.363	0.024	0.357	2.230	0.032	0.391	2.062	0.047	0.513	2.304	0.027	0.382	2.261	0.030	0.391
Ciprofloxacin	DM	Statistics	Holm *p*	DM	Statistics	Holm *p*	DM	Statistics	Holm *p*	DM	Statistics	Holm *p*	DM	Statistics	Holm *p*
RF	−1.919	0.063	0.632												
SVM	−1.866	0.070	0.634	−1.332	0.191	0.718									
XGB	−1.696	0.099	0.634	−0.969	0.339	0.718	0.577	0.568	0.718						
STACK-LM	1.370	0.179	0.718	1.765	0.086	0.634	1.813	0.078	0.634	1.667	0.104	0.634			
STACK-RF	2.577	0.014	0.201	2.435	0.020	0.262	2.357	0.024	0.290	2.221	0.033	0.362	2.842	0.007	0.112
Amoxicillin	DM	Statistics	Holm *p*	DM	Statistics	Holm *p*	DM	Statistics	Holm *p*	DM	Statistics	Holm *p*	DM	Statistics	Holm *p*
RF	−0.024	0.981	0.981												
SVM	1.045	0.303	0.746	2.321	0.026	0.210									
XGB	−1.447	0.157	0.627	−2.636	0.012	0.115	−3.135	0.003	0.049						
STACK-LM	2.083	0.045	0.312	2.066	0.046	0.312	1.173	0.249	0.746	2.886	0.007	0.080			
STACK-RF	2.921	0.006	0.079	2.667	0.012	0.115	2.078	0.045	0.312	3.245	0.003	0.039	2.849	0.007	0.080
Ceftriaxone	DM	Statistics	Holm *p*	DM	Statistics	Holm *p*	DM	Statistics	Holm *p*	DM	Statistics	Holm *p*	DM	Statistics	Holm *p*
RF	0.963	0.342	1.000												
SVM	0.918	0.365	1.000	−0.307	0.760	1.000									
XGB	0.734	0.468	1.000	−0.618	0.541	1.000	−0.318	0.752	1.000						
STACK-LM	1.074	0.290	1.000	0.662	0.512	1.000	0.694	0.493	1.000	0.890	0.380	1.000			
STACK-RF	1.542	0.132	1.000	1.138	0.263	1.000	1.172	0.249	1.000	1.279	0.209	1.000	1.259	0.216	1.000
Sulfamethoxazole	DM	Statistics	Holm *p*	DM	Statistics	Holm *p*	DM	Statistics	Holm *p*	DM	Statistics	Holm *p*	DM	Statistics	Holm *p*
RF	−1.395	0.172	0.690												
SVM	−1.262	0.215	0.690	−0.157	0.876	0.876									
XGB	−2.106	0.042	0.339	−2.380	0.023	0.252	−1.697	0.099	0.690						
STACK-LM	1.087	0.284	0.690	1.681	0.102	0.690	1.659	0.106	0.690	2.230	0.032	0.290			
STACK-RF	2.930	0.006	0.083	2.926	0.006	0.083	2.528	0.016	0.194	3.079	0.004	0.060	2.281	0.029	0.288
Azithromycin	DM	Statistics	Holm *p*	DM	Statistics	Holm *p*	DM	Statistics	Holm *p*	DM	Statistics	Holm *p*	DM	Statistics	Holm *p*
RF	0.246	0.807	1.000												
SVM	1.095	0.281	1.000	1.140	0.262	1.000									
XGB	−0.196	0.845	1.000	−0.491	0.627	1.000	−0.795	0.432	1.000						
STACK-LM	2.118	0.041	0.579	1.649	0.108	1.000	0.550	0.586	1.000	1.188	0.243	1.000			
STACK-RF	2.451	0.019	0.291	1.859	0.071	0.858	1.158	0.255	1.000	1.918	0.063	0.823	1.500	0.142	1.000
Doxycycline	DM	Statistics	Holm *p*	DM	Statistics	Holm *p*	DM	Statistics	Holm *p*	DM	Statistics	Holm *p*	DM	Statistics	Holm *p*
RF	−0.298	0.768	1.000												
SVM	0.315	0.754	1.000	0.648	0.522	1.000									
XGB	−1.202	0.237	1.000	−1.291	0.205	1.000	−1.500	0.142	1.000						
STACK-LM	1.431	0.161	1.000	1.438	0.159	1.000	0.757	0.454	1.000	1.699	0.098	1.000			
STACK-RF	1.580	0.123	1.000	1.612	0.116	1.000	1.319	0.196	1.000	1.758	0.087	1.000	1.541	0.132	1.000

**Table 4:** Leading XGBoost Gain values reported for the environmental and physicochemical predictors.

Antibiotic	Rank	Environmental and Physicochemical Predictor	Gain
Trimethoprim	1	COD	0.484
	2	pH	0.287
Ciprofloxacin	1	Turbidity	0.673
	2	Electrical conductivity	0.195
Amoxicillin	1	pH	0.255
	2	Water temperature	0.251
Ceftriaxone	1	Relative air humidity	0.612
Sulfamethoxazole	1	Turbidity	0.576
	2	pH	0.199
Azithromycin	1	BOD_5_	0.311
	2	COD	0.273
Doxycycline	1	Electrical conductivity	0.681
	2	Water temperature	0.267

**Table 5:** Performance metrics of machine-learning models for predicting antibiotic concentrations based on the occurrence profiles of culture-based presumptive resistant bacterial taxa.

Antibiotic	Model	R^2^	RMSE	MAE	AARD	SSE
Trimethoprim	ANN	0.012	220.453	133.154	3.8 × 10^12^	1.7 × 10^6^
	RF	0.009	199.670	136.197	4.3 × 10^12^	1.4 × 10^6^
	SVM	0.014	202.656	126.036	2.8 × 10^12^	1.5 × 10^6^
	XGB	0.006	255.550	163.905	5.4 × 10^12^	2.4 × 10^6^
	STACK-LM	0.240	165.071	113.743	3.6 × 10^12^	9.8 × 10^5^
	STACK-RF	0.876	91.650	59.635	2.0 ˑ 10^12^	3.0 × 10^5^
Ciprofloxacin	ANN	0.000	869.620	600.821	2.1 × 10^13^	2.7 × 10^7^
	RF	0.026	684.564	465.047	1.5 × 10^13^	1.7 × 10^7^
	SVM	0.002	682.674	447.170	1.1 × 10^13^	1.7 × 10^7^
	XGB	0.000	854.002	574.560	1.9 × 10^13^	2.6 × 10^7^
	STACK-LM	0.252	569.220	386.738	1.1 × 10^13^	1.2 × 10^7^
	STACK-RF	0.849	369.743	258.471	9.4 × 10^12^	4.9 × 10^6^
Amoxicillin	ANN	0.105	27.557	14.336	8.1 × 10^11^	2.7 × 10^4^
	RF	0.006	25.483	16.153	7.5 × 10^11^	2.3 × 10^4^
	SVM	0.000	25.691	14.066	4.7 × 10^11^	2.4 × 10^4^
	XGB	0.007	29.952	19.059	9.9 × 10^11^	3.2 × 10^4^
	STACK-LM	0.175	22.680	14.949	8.1 × 10^11^	1.9 × 10^4^
	STACK-RF	0.889	14.447	10.002	5.3 × 10^11^	7.5 × 10^3^
Ceftriaxone	ANN	0.003	90.204	32.958	1.8 × 10^12^	2.9 × 10^5^
	RF	0.006	66.482	27.553	1.3 × 10^12^	1.6 × 10^5^
	SVM	0.023	66.767	24.591	9.2 × 10^11^	1.6 × 10^5^
	XGB	0.003	82.579	33.197	1.8 × 10^12^	2.5 × 10^5^
	STACK-LM	0.147	59.802	30.036	1.8 × 10^12^	1.3 × 10^5^
	STACK-RF	0.945	31.913	13.564	6.7 × 10^11^	3.7 × 10^4^
Sulfamethoxazole	ANN	0.001	399.731	245.546	5.0 × 10^12^	5.8 × 10^6^
	RF	0.030	326.524	226.497	9.3 × 10^12^	3.8 × 10^6^
	SVM	0.000	332.979	205.700	4.8 × 10^12^	4.0 × 10^6^
	XGB	0.001	436.940	295.453	1.4 × 10^13^	6.9 × 10^6^
	STACK-LM	0.212	279.695	197.766	6.5 × 10^12^	2.8 × 10^6^
	STACK-RF	0.888	170.358	118.525	4.0 × 10^12^	1.0 × 10^6^
Azithromycin	ANN	0.014	76.403	37.413	6.2 × 10^11^	2.1 × 10^5^
	RF	0.000	51.795	26.110	5.5 × 10^11^	9.7 × 10^4^
	SVM	0.001	50.219	22.730	3.9 × 10^11^	9.1 × 10^4^
	XGB	0.000	63.938	36.104	8.7 × 10^11^	1.5 × 10^5^
	STACK-LM	0.024	48.197	25.283	4.4 × 10^11^	8.4 × 10^4^
	STACK-RF	0.819	30.551	15.797	2.3 × 10^11^	3.4 × 10^4^

**Table 6:** Pairwise comparison of predictive performance among ANN, RF, SVM, XGB, STACK-LM, and STACK-RF models for antibiotic concentration prediction using the Diebold–Mariano (DM) test with Holm-adjusted *p*-values.

	ANN			RF			SVM			XGB			STACK-LM		
Trimethoprim	DM	Statistics	Holm *p*	DM	Statistics	Holm *p*	DM	Statistics	Holm *p*	DM	Statistics	Holm *p*	DM	Statistics	Holm *p*
RF	0.942	0.353	1.000												
SVM	0.596	0.555	1.000	−0.216	0.831	1.000									
XGB	−2.092	0.044	0.350	−2.345	0.025	0.273	−1.671	0.104	0.516						
STACK-LM	1.767	0.086	0.516	2.008	0.052	0.366	1.756	0.088	0.516	2.388	0.022	0.270			
STACK-RF	2.813	0.008	0.112	2.609	0.013	0.173	2.224	0.033	0.295	3.091	0.004	0.058	2.318	0.026	0.273
Ciprofloxacin	DM	Statistics	Holm *p*	DM	Statistics	Holm *p*	DM	Statistics	Holm *p*	DM	Statistics	Holm *p*	DM	Statistics	Holm *p*
RF	1.909	0.064	0.406												
SVM	1.745	0.090	0.449	0.043	0.966	1.000									
XGB	0.158	0.875	1.000	−2.813	0.008	0.104	−1.960	0.058	0.406						
STACK-LM	2.225	0.033	0.296	1.680	0.102	0.449	1.466	0.152	0.455	2.399	0.022	0.241			
STACK-RF	3.261	0.002	0.037	2.636	0.012	0.149	2.269	0.030	0.296	3.076	0.004	0.057	2.129	0.040	0.323
Amoxicillin	DM	Statistics	Holm *p*	DM	Statistics	Holm *p*	DM	Statistics	Holm *p*	DM	Statistics	Holm *p*	DM	Statistics	Holm *p*
RF	0.439	0.663	1.000												
SVM	0.356	0.724	1.000	−0.192	0.849	1.000									
XGB	−0.584	0.563	1.000	−2.574	0.014	0.202	−1.999	0.053	0.488						
STACK-LM	1.217	0.232	1.000	1.109	0.275	1.000	1.006	0.321	1.000	2.133	0.040	0.480			
STACK-RF	2.041	0.049	0.488	2.473	0.018	0.239	2.091	0.044	0.482	3.101	0.004	0.057	1.952	0.059	0.488
Ceftriaxone	DM	Statistics	Holm *p*	DM	Statistics	Holm *p*	DM	Statistics	Holm *p*	DM	Statistics	Holm *p*	DM	Statistics	Holm *p*
RF	1.154	0.256	1.000												
SVM	1.106	0.276	1.000	−0.087	0.931	1.000									
XGB	0.395	0.695	1.000	−1.555	0.129	1.000	−1.360	0.182	1.000						
STACK-LM	1.301	0.202	1.000	1.210	0.234	1.000	0.874	0.388	1.000	1.655	0.107	1.000			
STACK-RF	1.673	0.103	1.000	1.431	0.161	1.000	1.291	0.205	1.000	1.860	0.071	1.000	1.435	0.160	1.000
Sulfamethoxazole	DM	Statistics	Holm *p*	DM	Statistics	Holm *p*	DM	Statistics	Holm *p*	DM	Statistics	Holm *p*	DM	Statistics	Holm *p*
RF	2.123	0.041	0.286												
SVM	1.659	0.106	0.424	−0.254	0.801	0.801									
XGB	−1.000	0.324	0.648	−2.919	0.006	0.079	−2.032	0.050	0.299						
STACK-LM	2.456	0.019	0.179	1.782	0.083	0.417	1.425	0.163	0.489	2.730	0.010	0.117			
STACK-RF	2.973	0.005	0.074	2.735	0.010	0.117	2.256	0.030	0.243	3.458	0.001	0.022	2.485	0.018	0.179
Azithromycin	DM	Statistics	Holm *p*	DM	Statistics	Holm *p*	DM	Statistics	Holm *p*	DM	Statistics	Holm *p*	DM	Statistics	Holm *p*
RF	1.509	0.140	1.000												
SVM	1.537	0.133	1.000	1.051	0.301	1.000									
XGB	0.980	0.334	1.000	−2.881	0.007	0.094	−2.737	0.010	0.126						
STACK-LM	1.620	0.114	1.000	1.964	0.057	0.632	0.967	0.340	1.000	2.907	0.006	0.094			
STACK-RF	1.962	0.058	0.632	1.460	0.153	1.000	1.259	0.216	1.000	2.308	0.027	0.325	1.302	0.201	1.000
Doxycycline	DM	Statistics	Holm *p*	DM	Statistics	Holm *p*	DM	Statistics	Holm *p*	DM	Statistics	Holm *p*	DM	Statistics	Holm *p*
RF	−0.298	0.768	1.000												
SVM	0.315	0.754	1.000	0.648	0.522	1.000									
XGB	−1.202	0.237	1.000	−1.291	0.205	1.000	−1.500	0.142	1.000						
STACK-LM	1.431	0.161	1.000	1.438	0.159	1.000	0.757	0.454	1.000	1.699	0.098	1.000			
STACK-RF	1.580	0.123	1.000	1.612	0.116	1.000	1.319	0.196	1.000	1.758	0.087	1.000	1.541	0.132	1.000

**Table 7:** Leading XGBoost Gain values for presumptive resistant bacterial-taxon predictors detected in wastewater and surface-water samples (Gain represents the relative improvement in XGBoost model performance attributable to a predictor. Only bacterial-taxon predictors with explicitly reported Gain values were included).

Antibiotic	Presumptive Resistant Bacterial-Taxon Predictor	Gain
Trimethoprim	*Aeromonas veronii*	0.128
	*Escherichia coli*	0.125
	*Citrobacter freundii*	0.081
	*Klebsiella pneumoniae*	0.058
Ciprofloxacin	*Citrobacter freundii*	0.136
	*Escherichia coli*	0.080
	*Stenotrophomonas maltophilia*	0.078
	*Aeromonas veronii*	0.076
Amoxicillin	*Klebsiella pneumoniae*	0.253
	*Citrobacter braakii*	0.107
	*Stenotrophomonas maltophilia*	0.093
Ceftriaxone	*Klebsiella pneumoniae*	0.519
	*Acinetobacter baumannii*	0.321
	*Serratia marcescens*	0.150
Sulfamethoxazole	*Klebsiella pneumoniae*	0.149
	*Aeromonas veronii*	0.105
	*Citrobacter freundii*	0.101
Azithromycin	*Escherichia coli*	0.277
	*Klebsiella pneumoniae*	0.055

## Data Availability

The original contributions presented in this study are included in the article and Supplementary Materials. Further inquiries can be directed to the corresponding author.

## Supplementary Materials

The following supporting information can be downloaded at: https://www.mdpi.com/article/10.3390/antibiotics15090920/s1, Table S1. Individual antibiotic concentrations in the supplied analytical dataset.
