# Supplementary material for: Explainable Stacked Ensemble Learning for Predicting Antibiotic Residues in the Danube River Within the Territory of the City of Novi Sad, Serbia
Source: Antibiotics (Basel). 2026 Sep 17;15(9):920. doi: 10.3390/antibiotics15090920 (PMC13603386; doi:10.3390/antibiotics15090920)
Supplement: Supplementary file 1 [file antibiotics-15-00920-s001.zip › Supplementary_Material.pdf]

Table S1. Individual antibiotic concentrations in the supplied analytical dataset

Type	Season	Trimethoprim	Ciprofloxacin	Amoxicillin	Ceftriaxone	Sulfamethoxazole	Azithromycin	Doxycycline
Wastewater	Summer	14.3	434.4	nd	nd	0	3.8	nd
		66.9	485.7	nd	nd	118.3	65.2	nd
		264	1696.9	nd	nd	692.6	23.9	132.2
		450.3	1311.6	nd	nd	763	38.3	nd
		422.4	513.5	nd	nd	580.9	7.1	108.2
		778.9	2122.5	nd	nd	1169.8	24.1	nd
		nd	80.1	nd	nd	nd	2.6	nd
		nd	nd	nd	nd	nd	nd	nd
		nd	nd	nd	nd	nd	nd	nd
Surface water		nd	43	nd	nd	nd	5.7	nd
		nd	35.7	nd	nd	nd	2.3	nd
		nd	91.8	nd	nd	nd	2	nd
		nd	67.3	nd	nd	nd	1.2	nd
		nd	63.7	nd	nd	2.6	1.6	nd
		nd	nd	nd	nd	0	nd	nd
		nd	nd	nd	nd	3.8	1.7	nd
		nd	nd	20.9	nd	2.9	2.1	nd
		nd	nd	nd	nd	nd	nd	nd
Wastewater	Autumn	26.5	243.1	nd	184	158.7	nd	nd
		145.5	949	nd	nd	74.1	273.1	nd
		204.8	934.6	81.4	353.3	310.2	23.4	nd
		228.8	1533.6	87	nd	575	36	nd
		453.8	721	nd	nd	439.1	116.3	nd
		522.1	2534.3	84.5	nd	1064.6	45	nd
		nd	31.4	58.6	36.6	nd	9.6	nd
		nd	nd	nd	nd	nd	nd	nd
		nd	nd	nd	nd	nd	nd	nd
Surface water		10.4	nd	nd	nd	3.2	1.7	nd
		10.1	nd	nd	nd	3.3	1.5	nd
		7.1	nd	nd	nd	2.6	1.4	nd
		6.2	nd	nd	nd	3.2	1.1	nd
		6.5	nd	nd	nd	2.7	1.4	nd
		nd	nd	nd	nd	nd	nd	nd
		6.6	0	0	0	2.5	1.2	0

	8.8	0	29.5	0	3.5	1.4	0
	0	0	0	0	0	0	0
